# AND-logic MRI contrast by water flux modulation

**DOI:** 10.1039/d6sc01039c

**Published:** 2026-05-14

**Authors:** James P. Smith, Connor M. Ellis, Anna M. Duncan, Jason J. Davis

**Affiliations:** a Department of Chemistry, University of Oxford South Parks Road Oxford OX1 3QZ UK Jason.davis@chem.ox.ac.uk +44 (0)1865 275 914

## Abstract

Stimuli-responsive MRI agents enable disease-specific imaging by selectively modulating generated contrast in pathological microenvironments. To date, these moieties have been largely limited to those that respond to one specific (potentially false positive) trigger. This work reports AND-logic gated silica nanoparticles modified with a pH-responsive poly(2-(dimethylamino)ethyl methacrylate) (pDMAEMA) shell, tethered to the particles *via* a reducible 4,4′-diaminoazobenzene (AZO) cross-linker. These nanoparticles support very high levels of contrast switching exclusively under concurrent mildly acidic and reducing conditions, which are hallmarks of solid tumours, myocardial ischemia, and chronic inflammation.

## Abstract 2

In this work we demonstrate that relaxivity, and by extension image contrast, can be modulated by an AND-logic polymeric gate at a mesoporous silica interface that is responsive to both low pH and hypoxic microenvironments.

## Graphical abstract

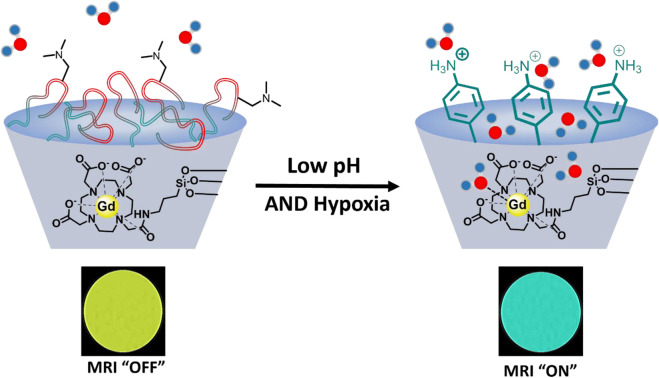

## Introduction

Magnetic resonance imaging (MRI) is a powerful non-invasive imaging technique widely used by clinicians to detect and monitor a broad range of pathological conditions, due to its high spatial resolution and deep tissue penetration.^1^ However, the inherently low signal-to-noise ratio arising from the relatively small variation in longitudinal relaxation times (*T*_1_) across soft tissues can limit diagnostic clarity, leading to poor tissue delineation and reduced image contrast.^2^ Paramagnetic contrast agents (CAs), commonly based on Gd^3+^ chelates, can mitigate this limitation by increasing the baseline longitudinal relaxation rate. Their efficacy is quantified by the longitudinal relaxivity, *r*_1_ (defined as Δ(1/*T*_1_)/[CA]).^3^ High relaxivity values require a lower [CA], and therefore a lower administered dose of clinical agent, to generate effective and observable improvements in image contrast.

Prior work has shown that Gd-doped mesoporous silica nanoparticles (GdMSNs) constitute a synthetically tuneable and biocompatible CA capable of delivering substantial *T*_1_ contrast.^4–6^ Their amenability to post-synthetic modification with organic or inorganic–organic hybrid functionalities allows the incorporation of peripheral motifs such as acids, bases, or labile intra- and supramolecular interactions, conferring pronounced environmental responsivity. Contrast modulation can be triggered by endogenous or exogenous stimuli, including pH, temperature, light exposure, or enzymatic activity.^7,8^ Such stimuli-mediated responses are attractive for disease-specific reporting, facilitating targeted and enhanced diagnostic imaging.^9^

For paramagnetic nanoparticulate contrast agents, *r*_1_ is determined by the accessibility and mobility of water molecules (residence time *τ*_M_, and diffusional correlation time *τ*_D_)^10^ in close proximity to the metal centres. When these centres are embedded in mesopores we have shown that a particle-peripheral polymeric surface capping gates water access and so *r*_1_.^11^ When the polymer is collapsed, the gate is closed, and the contrast generated becomes limited by the mesopore water volume (which fully relaxes on a timescale that is short relative to the interval between radiofrequency pulses). Under conditions where the polymer conformation switches to that of an extended brush, water exchange with bulk is enabled and *r*_1_/contrast grows markedly.

In the design of targeted nanoparticle theranostics, redox-sensitive moieties have attracted considerable interest, as reducing microenvironments represent a hallmark of numerous pathological states, including cancer and cardiomyopathy. The overproduction of endogenous reductants is both a consequence of, and a contributor to, the disease pathogenesis.^12,13^ A range of redox-labile functionalities, such as disulfide linkages, have, accordingly, been incorporated into molecular contrast agents to enable environment-specific imaging.^14^ These efforts, however, have largely focused on small-molecule systems (of low relaxivity). Translation to much higher contrast supporting nanoparticulate agents has, thus far, been limited to studies of triggered aggregation/dispersion with *T*_2_-active superparamagnetic entities.^15^

**Fig. 1:**
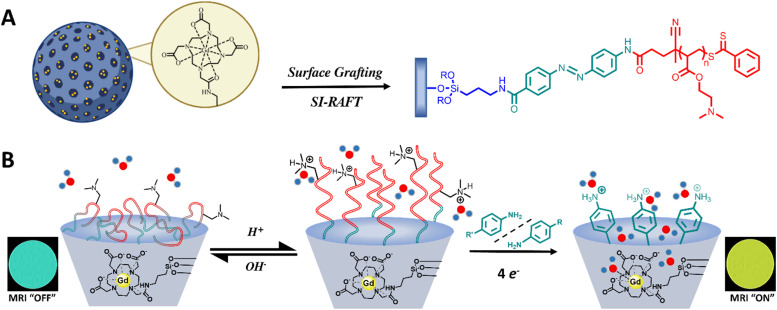
Schematic depiction of the generation of AND-logic gated MR contrast nanoparticulate scaffolds. (A) Surface-initiated reversible addition–fragmentation chain-transfer (SI-RAFT) enables surface grafting of a pDMAEMA brush to the MSN surface. Successive modifications with an aminated silane (APTES), succinic anhydride, AZO, and the chain transfer agent (CTA) yields the desired particles. (B) Upon simultaneous exposure to acid and reductant, tertiary amine protonation facilitates polymer expansion, reagent access, and reductive cleavage of the azo (N=N) bonds, releasing the surface tethered polymer (degree of polymerisation, *D*_P_ ≈ 23). This, in turn, switches on diffusive water access to the MSN pore-confined Gd-chelates. Reductant access to the azo bonds is enabled only at low pH. Upon meeting these specific conditions, MRI contrast is fully activated.

In considering the known reductive cleavability of disulfide linkers,^16^ we posited that the tethering of the polymer *via* a reduction-labile azobenzene (AZO, N=N) linkage would engender a two-stage activation (Fig. 1A). Significantly, cleavage of the azobenzene bond is contingent upon physical access of a reductant to the linker, introducing an additional level of control beyond simple redox sensitivity (Fig. 1B). Under pathological reducing conditions, such as elevated concentrations of nicotinamide adenine dinucleotide phosphate (NADPH), azoreductase (AzoR) enzymes present in hepatic carcinomas, or glutathione (GSH),^17^ reductant access to the AZO linkage was envisaged to induce its cleavage (and removal of the water-gating polymer), thereby restoring diffusive water exchange to the MSN-internalised Gd-chelates. When the polymer scaffold is itself responsive, we introduce AND-logic behaviour, where reductant access to the internal AZO moieties is additionally regulated. Only under conditions where the polymer is sufficiently protonated, and expanded, to permit reductant ingress does AZO cleavage occur. This dual-trigger requirement restricts activation to microenvironments that are both acidic and reducing, enhancing pathological specificity and reducing the likelihood of false-positive contrast activation.

Poly(2-(dimethylamino)ethyl methacrylate) (pDMAEMA) was herein selected as the responsive polymer, as its associated p*K*_a_ (6.5–7.4)^18^ falls within a clinically useful range (5.8–7.0),^19^ relevant to the extracellular fluid of tumours and atherosclerotic lesions.^20–22^ We have previously shown within nanoparticulate systems that protonation-dependent swelling and deswelling of post-synthetically applied polymers is able to gate water and reversibly modulate water access.^11,23,24^ It is known that polymer-integrated azobenzene motifs can support hypoxia-responsive drug release under physiologically relevant acidic and reductive conditions associated with cancer.^25–28^ We have, then, combined these principles to generate pDMAEMA-AZO-GdMSNs that respond selectively under the clinically-relevant concurrent acidic and reductive conditions.^29^ Paramagnetic GdMSNs were functionalised at the surface and outer pores with a highly hydrophobic AZO, (initially limiting water exchange into the pores), to which the pH-responsive pDMAEMA shell was subsequently tethered. This architecture features a highly suppressed MRI ‘off-state’, owing to the co-operative steric and diffusional water gating effects imparted by the globular polymer cap and the hydrophobic azobenzene linker. At a pH > p*K*_a_ of the polymer, the deprotonated pDMAEMA adopts a globular conformation that sterically blocks reductant access to the particle surface and pores. At pH < p*K*_a_, protonation of the polymer increases hydrophilicity, enabling an influx of reductant-carrying solvent to the inner AZO. Only under these dual-stimuli conditions does polymer cleavage occur such that water exchange between bulk and particle-internal paramagnetic ion is enabled, yielding a very marked enhancement in relaxivity.

This strategy was additionally translated to hollow MSNs (HMSNs) encapsulating the clinically employed molecular contrast agent Gd-DOTA (gadolinium(iii) 1,4,7,10-tetraazacyclododecane-1,4,7,10-tetraacetate, Dotarem®, freely diffusing and not side wall tethered). It was envisaged that the steric gating imparted by the polymer shell could be extended to regulate the release of these, initially, MRI-silent chelates (used in about 40% of all clinical MRI scans). The principles demonstrated herein are, of course, readily adaptable to a broad range of cargos, either in place of or alongside Gd-DOTA, offering clear potential for targeted cancer theranostic applications.^30^

## Results and discussion

### Mesoporous silica nanoparticles

GdMSNs with chelates localised to the interior of the pores were synthesised by a modified Stöber method previously reported by the group.^23^ The particles displayed a high relaxivity of 27.62 ± 0.43 mM^−1^ s^−1^ and a dynamic light scattering (DLS) resolved hydrodynamic size of 107.6 ± 2.6 nm with high uniformity and low polydispersity (PDI < 0.1).^31^ Particle pore size was determined to be 3.2 ± 0.3 nm (BJH pore size analysis, SI 2). The particles were aminated with 0.3 mol% (with respect to the silanol architecture) of (3-aminopropyl)triethoxysilane (APTES) biased to the outer mesopores, controlled at the point of synthesis through time-delayed amination.^30^ Early addition of APTES (<10 minutes) biases the amines towards the inner pores as the particles begin forming, whereas later addition (30–60 minutes) biases amines towards the outer pores and surface.^31^ Reaction of integrated amines with DOTA-NHS ester, and subsequent metalation with Gd^3+^ (see SI), afforded the desired GdMSNs. A colourimetric assay using arsenazo(iii) confirmed the nearly undetectable presence of unchelated Gd^3+^ adsorbed to the particles, contributing at maximum 0.51% of total relaxivity (0.1765 µM, 0.005%, SI 1).

**Fig. 2:**
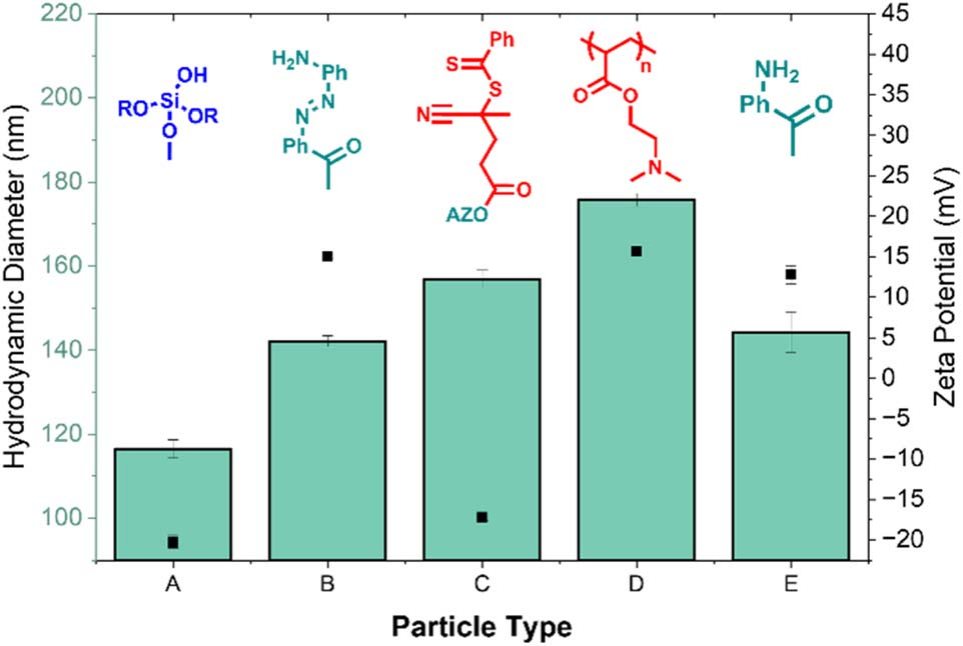
DLS characteristics of various particles generated in the synthetic steps towards pDMAEMA-AZO-GdMSNs, and the final, reductively cleaved product. Chemical structures of the dominant surface chemistry that influences the measured properties are shown. Particles are (A) native GdMSNs, (B) AZO-GdMSNs, (C) CPADB-AZO-GdMSNs, (D) pDMAEMA-AZO-GdMSNs, and (E) pDMAEMA-AZO-GdMSNs after exposure to AND-logic conditions.

Surface functionalisation with the CTA, 4-cyano-4-(phenylcarbonothioylthio)pentanoic acid (CPADB) and AZO was achieved *via* successive coupling steps (Fig. 1A). The GdMSN surface was first modified post-synthetically with APTES, followed by conversion of the amines to carboxylic acids using succinic anhydride. The azobenzene linker was integrated *via* amide coupling and subsequently reacted with activated CPADB-NHS to yield CPADB-AZO-GdMSNs. Although the CPADB-AZO moiety has an optimised molecular diameter of 2.35 nm (SI 3; slightly smaller than the BJH derived pore diameter) and thus there could be some degree of internal pore, as well as outer surface, functionalisation. However, the steric constraints around this are unlikely to support polymerisation, therefore it can be assumed that polymer strands originate from the particle surface. DLS analyses were consistent with expectations (Fig. 2). Modification to the outer pore and particle surface with anionic or neutral moieties (COOH and CPADB) yielded negative *ζ*-potentials (COO^−^ and Si–O^−^), with AZO modifications generating positive *ζ*-potentials 
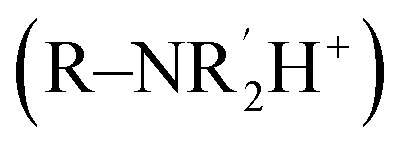
. Ultraviolet-visible spectroscopy (UV-vis, SI 4) was used to verify AZO and CPADB integration, and to calculate the associated grafting densities of 1.88 and 1.86 groups per nm^2^ of AZO and CTA, respectively (99% conversion of AZO to CPADB-AZO).^32^ Silanols reach a maximal density of approximately 4.6 per nm^2^ in fused silica; with 1.86 CTA per nm^2^ representing a densely functionalised surface.^33^ The reducible CPADB-AZO silane precursor was synthesised to validate the nanoparticulate synthetic conditions. It was derived from an AZO analogue, CPADB, and APTES through successive amide couplings (synthetic details in SI pages 4–5).^34^

Subsequent polymerisation with DMAEMA yielded a hydrophobic coating with an according size increase of 19.0 ± 3.7 nm. Polymer breathing was quantified by DLS, showing reversible hydrodynamic size changes in response to p*K*_a_ traversal (SI 10). Polymerisation was quantitatively analysed by thermogravimetric analysis (TGA, SI 8), with a resolved 35% mass loss at 700 °C, in line with calculations for the measured grafting density and degree of polymerisation (SI 17).^23^ Attenuated total reflectance infrared spectroscopy (ATR-IR, SI 9), quantitative NMR spectroscopy (showing monomer conversion over time, SI 7), and negatively stained transmission electron microscopy (TEM, SI 6) further verified peripheral surface modifications and polymer growth. Reductive AZO cleavage was confirmed with ^1^H NMR analysis (SI 13) of the collected residue and the residual particle *ζ*-potential (13.6 ± 1.7 mV; R–Ph–NH_2_/NH_3_^+^).

The exceptional water-gating ability of the pDMAEMA-AZO-GdMSNs resulted in a ∼85% collapse in *r*_1_ compared to a native GdMSN.^35^ Previous work from the group has demonstrated that a pDMAEMA brush alone is insufficient to heavily modulate water exchange by itself.^23,36,37^ Only upon introduction of additional functionality can a pDMAEMA brush affect water exchange. The azobenzene cross-linker in the present system enables this pronounced relaxivity suppression, implicating the highly hydrophobic nature of AZO as integral. The azobenzene moiety exhibits structural and physicochemical similarities to hydrophobic azo dyes such as aniline yellow (octanol/water partition coefficient, log *P* ≈ 4.2).^38^ Its integration here, then, dramatically reduces hydration of the surface and mesopore entrances, thereby further suppressing water exchange with exterior bulk. Within this architecture, the pDMAEMA brush serves two synergetic roles in generating AND-logic: it stabilises the hydrophobic nanoparticles in aqueous media and, when in its collapsed conformation at pH > p*K*_a_, it sterically restricts reductant access to the AZO linkage, as shown by UV-vis analysis of single- and dual-stimulus exposure (SI 11).

The effects of AZO functionalised polymer grafting density were investigated by employing a parallel ‘graft-to’ approach whereby a pre-synthesised and modified AZO-pDMAEMA was coupled to the particle surface to the aforementioned ‘graft-from’ (SI-RAFT) approach.^39^ The graft-to method typically produces lower grafting densities due to kinetic constraints associated with attaching large polymer chains to confined surfaces such as silica nanoparticles.^40^ Consistent with this, both relaxivity suppression and switching magnitude were attenuated (SI 12), compared to observations with the SI-RAFT particles, which were, thereafter, taken forwards as the preferred compositions for subsequent studies.

**Fig. 3:**
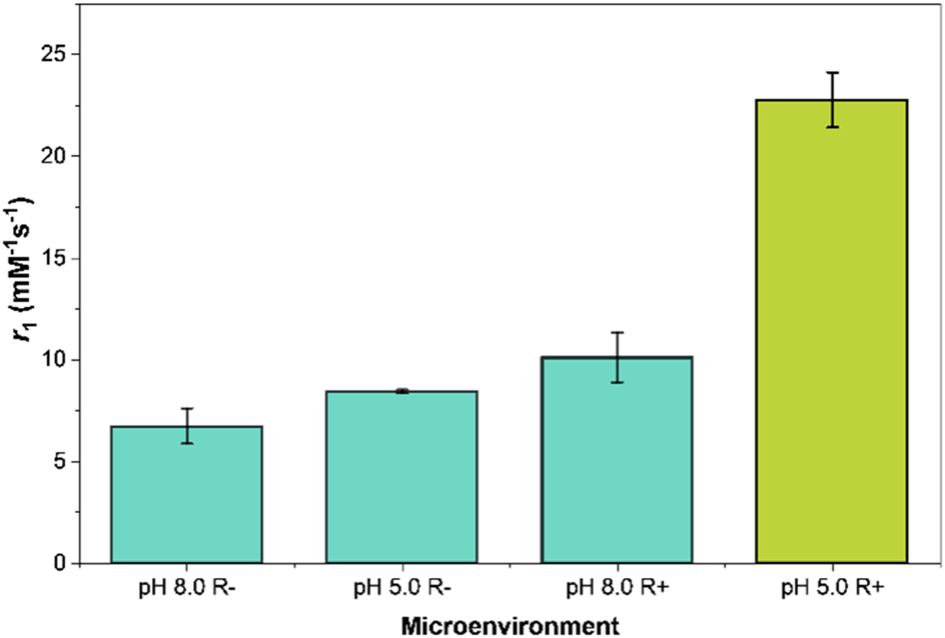
Relaxometric properties of the pDMAEMA-AZO-GdMSNs at 1.41 T and 298 K in a pH 8.0 (basified with 1.0 M NaOH) and non-reducing environment (pH 8.0 R−), a pH 8.0 and reducing environment (pH 8.0 R+, 2.0 mM Na_2_S_2_O_4_), a pH 5.0 (acidified with 1.0 M HCl) and non-reducing environment (pH 5.0 R−), and a pH 5.0 and reducing environment (pH 5.0 R+). The suppressed relaxivity values prior to dual-stimuli exposure highlights the synergistic effects of the AZO-pDMAEMA coating.

The relaxivities of the (SI-RAFT) pDMAEMA-AZO-GdMSNs were evaluated at both pH = 5.0 without the 2.0 mM sodium dithionite reductant (R−), and at pH = 8.0 with reductant (R+). Na_2_S_2_O_4_ was used as an accepted small molecule mimic for biological reduction by AzoR/NADPH, providing an effective model for hypoxic cellular conditions.^41^ The reductant concentration was chosen to lie in a range relevant to both hypoxic mimicking and the [AZO] concentration (0.15 mM per 1 mg mL^−1^ particle suspension).^42^ This was specifically to probe the relationship between polymer conformation, the steric gating of reductants, and water exchange into the mesopores (Fig. 1B).^43^ The particles exhibited clear AND-logic behaviour: modest relaxivity enhancements of approximately 25% and 50% were observed in response to acidic and reducing stimuli alone, respectively (Fig. 3, pH 5.0 R− and pH 8.0 R+). The larger relaxivity increase observed upon exposure to reductant alone (Δ*r*_1_ = 3.36 ± 2.10 mM^−1^ s^−1^; 50%) suggests that a fraction of azobenzene linkers remains accessible to the reductant even when pDMAEMA adopts a collapsed globular conformation under basic conditions (*i.e.*, at pH ≈ 8.0).^44^ Exposure to pH 5.0 and 2.0 mM Na_2_S_2_O_4_ simultaneously results in a very pronounced (238%) increase in longitudinal relaxivity (Fig. 3). The passivating polymer coat, and surface AZO functionalisation very clearly drastically limits the ability of the internalised water pool to undergo exchange with bulk local to the pores. Successful reductive cleavage under these conditions was supported by size and *ζ*-potential measurements (SI 6), indicative of aniline (log *P* = 0.9) and its protonated form on the particle surface.

The effects of enhanced water exchange can be simulated by modulating an “effective *τ*_M,_” with realistic adjustments to this parameter reproducing the observed experimental trends (SI 16.2). Under conditions where access to bulk water is restricted, the local water pool becomes limited, increasing the likelihood that the chelate interacts with previously relaxed water molecules. Upon removal of the polymer barrier, and restoration of access to bulk solution, the paramagnetic centre can more efficiently and continuously exchange with perturbed water molecules, resulting in enhanced relaxivity as described by the proposed framework of “effective *τ*_M_”.

**Fig. 4:**
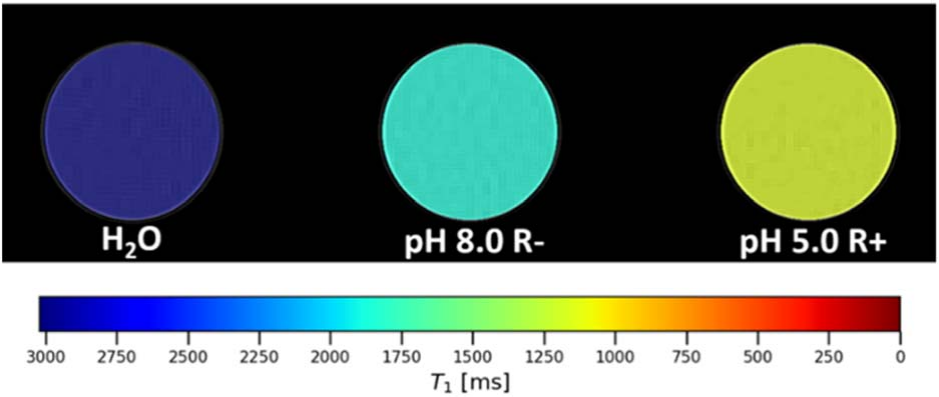
A colourised phantom MRI *T*_1_ of water, pH 8.0 R−, and pH 5.0 R+ taken at 4.7 T, 298 K shows the correlated decreases to the *T*_1_ times (3020 ± 15, 1874 ± 19, and 1142 ± 4 ms, respectively). Raw MRI phantom images can be found in SI 14.

The concurrent conditions required to facilitate gate removal were further confirmed by analyses with a 4.7 T (298 K) pre-clinical MRI scanner. Resolved phantom images (Fig. 4, raw images SI 14) showed marked and predictable differences in image contrast (*ca.* 1 mg mL^−1^) in images generated from a 30 minute scan. Exposure to concurrent stimuli yielded an increase to 1/*T*_1_ of 58%, in line with the 64% increase measured *via* NMR at 1.41 T.

**Fig. 5:**
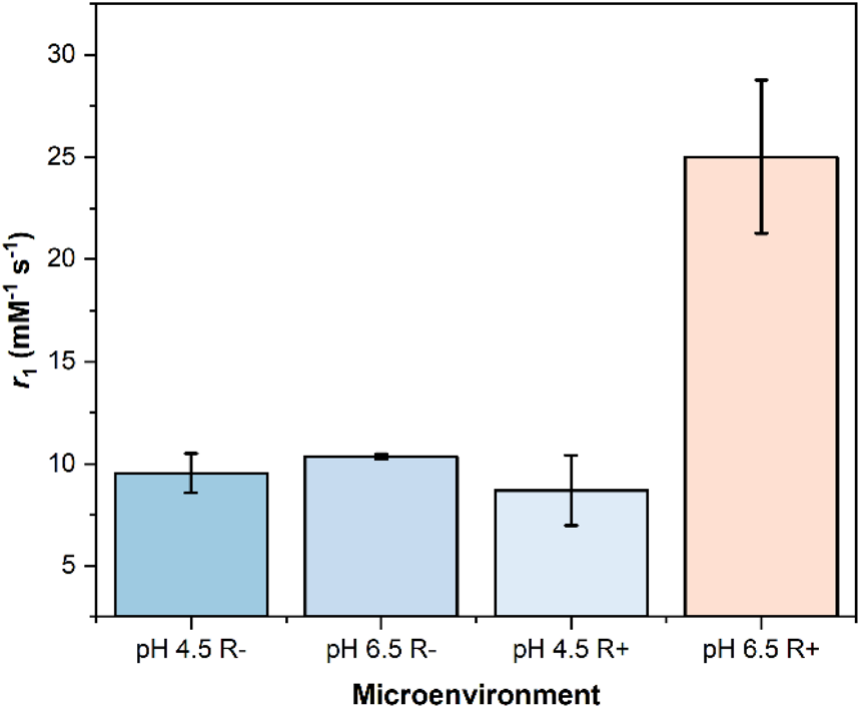
Relaxometric properties of the pMAA-AZO-GdMSNs at 1.41 T and 298 K in a pH 4.5 (acidified with 1.0 M HCl) and non-reducing environment (pH 4.5 R−), a pH 4.5 and reducing environment (pH 4.5 R+, 2.0 mM Na_2_S_2_O_4_), a pH 6.5 (acidified with 0.10 M HCl) and non-reducing environment (pH 6.5 R−), and a pH 6.5 and reducing environment (pH 6.5 R+). The observed values reflect the potent water exchange suppression supported by the AZO-pDMAEMA coating, with relaxivity restored upon reductive cleavage as the polymer swells.

To demonstrate the generality of this approach and to directly probe the role of polymer hydration and its influence on reductant accessibility in the observed gating behaviour, poly(methacrylic acid) (pMAA) was employed as a comparative responsive polymeric coating using an identical azobenzene-based tethering strategy. pMAA is widely used in pH-responsive delivery systems owing to its physiologically relevant p*K*_a_ (5.2).^45,46^ In its deprotonated form, pMAA exhibits strong hydrogen-bonding interactions with water.^47^ Despite this intrinsically hydrophilic character, low relaxivity values (*r*_1_ ≈ 9 mM^−1^ s^−1^) were observed when the particle was exposed to either base or reductant alone (AND-logic conditions not met), highlighting the importance of the hydrophobic azobenzene gate at the particle surface. Upon concurrent base and reductant exposure, a significant switch (Δ*r*_1_ = 162%) in relaxivity was observed, consistent with cleavage of AZO and restoration of water exchange (Fig. 5). It is worth noting that the *r*_1_ value at pH 6.5 (10.36 ± 0.14 mM^−1^ s^−1^), when the pMAA is in the extended conformation is markedly lower than that of a pMAA-GdMSN lacking azobenzene cross-linking previously reported by the group (*r*_1_ ≈ 49 mM^−1^ s^−1^ at 1.41 T). This, again, highlights the pivotal role of the internal hydrophobic AZO gate.

### Hollow mesoporous silica nanoparticles

Hollow mesoporous silica nanoparticles (HMSNs) possessing a large internal void suitable for encapsulation of molecular contrast agents were synthesised to evaluate the efficacy of the polymer coating in gating paramagnetic cargo release (as opposed to molecular water flux).^48,49^ The substantial internal volume of these particles facilitates efficient loading of a wide range of cargos, with potential broad applications.^50^ HMSNs were prepared using an established core–shell selective etching strategy.^51^ Briefly, solid silica cores were first generated (see SI 1.3.1 for synthetic details), followed by deposition of a mesoporous silica shell using the modified Stöber method described above. Selective removal of the solid core was then achieved through mild alkaline etching, and extraction of the surfactant template was performed under reflux in acidified ethanol (1 : 30 v/v HCl : EtOH), yielding the desired hollow architecture (SI 13). The resulting HMSNs possessed an average hydrodynamic size of 151.1 ± 1.4 nm and *ζ*-potential of −38.8 ± 0.4 mV, in line with previous reports from literature, with low polydispersity (PDI = 0.13).^52^ TEM provided direct visualisation of the expected morphology, clearly distinguishing the electron-dense porous silica shell from the low-contrast hollow interior (Fig. 8).

**Fig. 6:**
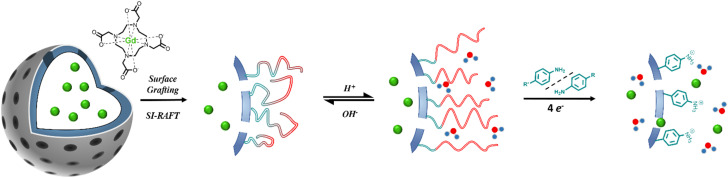
The surface chemistry of the synthesised pDMAEMA-AZO-HMSNs loaded with Gd-DOTA. In either acidic or reducing conditions alone, the bound polymer restricts the diffusion of both water and Gd-DOTA from/to bulk solution. Upon reductive cleavage, the entrapped chelate is released into the bulk, removing the limit on relaxivity as imposed by the reduced water pool initially available.

Surface functionalisation with CPADB-AZO was carried out using identical methods to those employed for the solid GdMSNs. The resulting CPADB-AZO-HMSNs were subsequently loaded with Gd-DOTA by incubation in a 30 mM Gd-DOTA DMF/water solution prior to SI-RAFT polymerisation of DMAEMA (Fig. 6, SI 1.3.1). External Gd-DOTA was removed with successive washings with solvents that retained the collapsed conformation of pDMAEMA. DLS revealed a hydrodynamic diameter and *ζ*-potential consistent with pDMAEMA grafting (225.3 ± 12.1 nm, 35.4 ± 4.7 mV), while TGA showed 21% mass loss at 700 °C (without Gd-DOTA loading), with decreased polymer mass reflective of the increased proximity of radicals within the pores and internal cavity, leading to increased odds of termination, and slower kinetics.^53^ ICP-MS measurements of the supernatant of a 1 mg mL^−1^ suspension over time showed negligible cargo release, prior to particle exposure to triggers (see below and SI 15).

The pDMAEMA-AZO-HMSNs exhibited strong relaxivity suppression (*r*_1_ = 1.05 ± 0.02 mM^−1^ s^−1^) compared to free Gd-DOTA (*r*_1_ = 3.80 mM^−1^ s^−1^) at 1.41 T and 298 K, attributed to confinement of a relatively small internal water pool with a large quantity of encapsulated contrast agent (1.07 × 10^8^ water molecules *vs.* 5.51 × 10^4^ Gd-DOTA in an ideal spherical HMSN interior of radius 45 nm), analogous to observations in previously reported liposomal contrast agents.^54^ Under these conditions, a single Gd-DOTA can interact with 1.0 × 10^7^ water molecules per second in the inner sphere (*τ*_M_ = 97 ns),^55^ or 5.7 × 10^11^ water molecules per second for the paramagnetic cargo as a whole; 3 magnitudes more than the quantity of water available within the cavity. Since practically all local water protons will, then, be relaxed shortly after the 180° RF pulse is applied, relaxivity/contrast generation is initially highly suppressed.

**Fig. 7:**
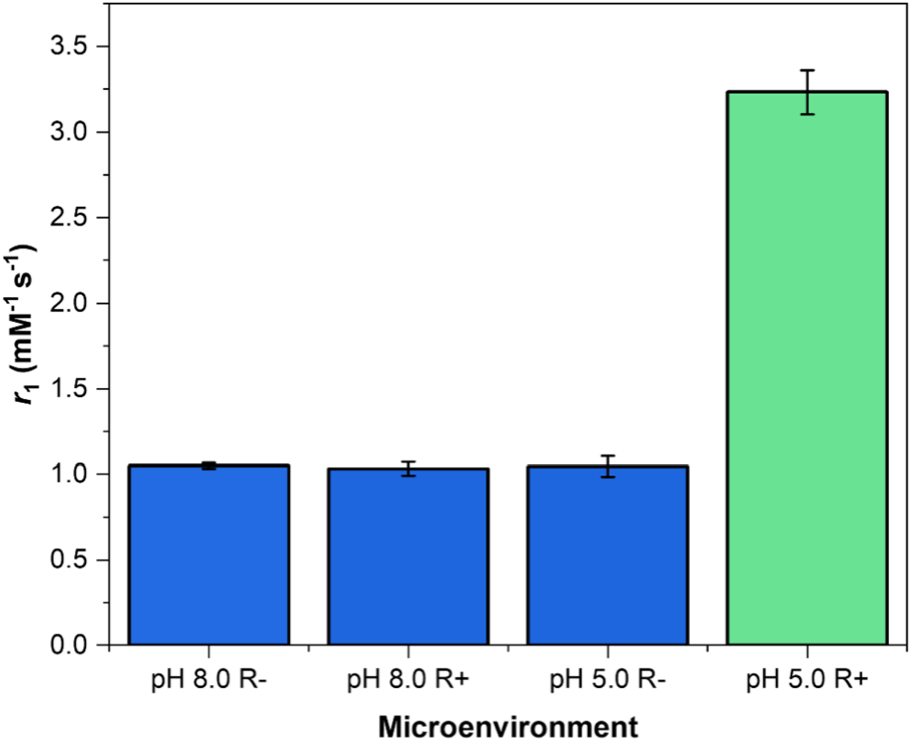
Relaxivity assessments of the synthesised HMSNs doped with Gd-DOTA at 1.41 T and 298 K in a pH 8.0 (basified with 1 M NaOH) and non-reducing environment (pH 8.0 R−), a pH 8.0 and reducing environment (pH 8.0 R+, 2.0 mM Na_2_S_2_O_4_), a pH 5.0 (acidified with 1 M HCl) and non-reducing environment (pH 5.0 R−, HCl), and a pH 5.0 and reducing environment (pH 5.0 R+). Longitudinal relaxivity values demonstrated the logic-gating effect. Relaxivity remains stable and suppressed prior to complete reductive cleavage, as gating the release of the paramagnetic cargo plays a larger role in the observed shifts in this case. As [Gd]/[H_2_O] is much larger, small changes to water flux do not significantly impact relaxivity. The cargo must be released from the interior to restore contrast. Gating of the larger Gd-DOTA (0.871 nm) is more effective than that of water (0.275 nm).^56^

As observed in the solid MSN analogue, exposure to either reductive (2.0 mM Na_2_S_2_O_4_) or mildly acidic conditions (pH 5.0) alone maintained suppression of both water diffusion and cargo release. Longitudinal relaxivity assessments exhibited significant (>200%) change only on concurrent exposure to both stimuli (Fig. 7). The AND logic gating of paramagnetic chelate release (∼90% of the payload, 2.62 × 10^16^ Gd-DOTA per mL) was confirmed by ICP analyses of the supernatant (SI 15). Phantom images captured using a 4.7 T clinical MRI scanner at 298 K (Fig. 9, SI 14) mirrored results collected *via* NMR measurements.

**Fig. 8:**
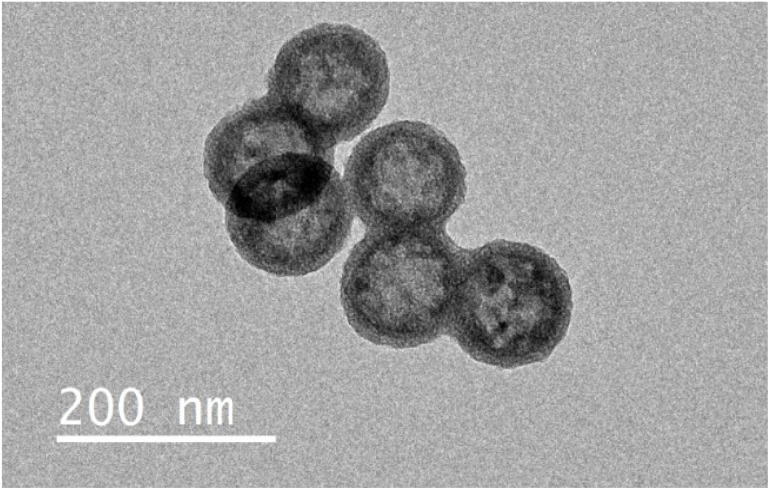
TEM imaging of HMSNs showing the characteristic electron dense outer shell, with a lighter hollow interior.

**Fig. 9:**
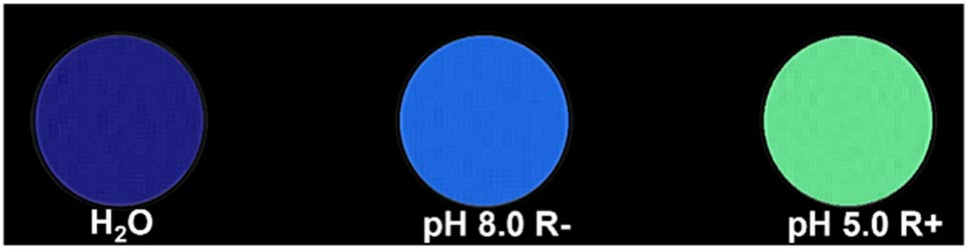
A colourised *T*_1_ map of the samples taken at 4.7 T and 298 K is in agreement with the observed relaxivities (*T*_1_ times are 3020 ± 15, 2740 ± 10, and 2426 ± 11 ms, respectively). Raw phantom MRI images are available in SI 14.

Despite its widespread clinical use, the standard administration of Gd-DOTA results in its systemic distribution.^57^ Its silencing and environmentally triggered release (potentially concurrently with a high payload of therapeutic) is both novel and of value on considering derived diagnostic–therapeutic (theranostic) platforms.

## Conclusions

Responsive polymer coatings provide a versatile means to tailor inorganic nanoparticle frameworks through stimulus-driven modulation of peripheral hydrophilicity. The distinct conformations adopted by such polymers impart steric control over the ingress and release of small molecules within mesoporous architectures. Applying these principles, the AND-logic gated pDMAEMA/pMAA-AZO-GdMSNs reported herein exhibit exceptionally large relaxivity switching (Δ*r*_1_ > 200%), while maintaining minimal off-target activation (<50% response to a single stimulus). This design could enable disease-specific imaging while minimising off-target activation, a significant advance over previous single-stimulus systems.^9^

The sequential and co-operative responses of the peripheral pH-responsive polymer and the reductively cleavable azobenzene linkage produce the observed large-amplitude relaxivity changes, aided by the strongly suppressed initial “off-state.” Future studies could explore alternative cross-linkers (*e.g*., light, oxidation, or enzyme-sensitive motifs), as well as chemical modification of AZO. Tuning the electronic properties of the cross-linker would enable control over both its susceptibility to biological reductants (*e.g*., GSH) and the rate of reduction.^58^ In addition, expansion of the polymer library to include block or mixed polyelectrolyte systems could enable tuning of p*K*_a_ values to align the pH-dependent response with the range of acidity observed across different disease states. Incorporation of reduction-sensitive motifs within the polymer itself may further enable control over time-dependent activation and enzyme-specific responsiveness. Such developments would enhance the modularity of this platform for tailored diagnostic applications.

## Author contributions

J. P. S. synthesised all particles and performed data acquisition. C. M. E. collected MRI images and assisted with experimental design. A. M. D. contributed to experimental design and theory. J. J. D. conceptualised and designed the project. All authors contributed to the writing of the manuscript.

## Conflicts of interest

There are no conflicts to declare.

## Supplementary Material

SC-017-D6SC01039C-s001

## Data Availability

The data supporting this article have been included as part of the supplementary information (SI). Supplementary information is available. See DOI: https://doi.org/10.1039/d6sc01039c.
