# Supplementary material for: AND-logic MRI contrast by water flux modulation
Source: Chem Sci. 2026 May 14;17(25):12589–96. doi: 10.1039/d6sc01039c (PMC13187857; doi:10.1039/d6sc01039c)
Supplement: SC-017-D6SC01039C-s001 [file SC-017-D6SC01039C-s001.pdf]

Supplementary Information (SI) for Chemical Science.
This journal is © The Royal Society of Chemistry 2026

Supplementary Information

AND-Logic MRI Contrast by Water Flux Modulation

James P. Smith [a], Connor M. Ellis [a], Anna M. Duncan [a], and Jason J. Davis[*a]

a. Department of Chemistry, University of Oxford, South Parks Road, Oxford, OX1 3QZ, UK. E-mail: Jason.davis@chem.ox.ac.uk; Tel: +44 (0)1865 275 914

1. Experimental

1.1 Materials

Triethanol amine (TEA) was purchased from Scientific Laboratory Supplies. 37% hydrochloric acid (HCl), N,N-dimethylformamide (DMF) and 70% nitric acid (HNO_3) were purchased from Fischer Scientific. DOTA-NHS ester was purchased from CheMatech. All other chemicals were purchased from Merck and used as received. Ultrapure water (Millipore) with a resistivity of 18.2 MΩ·cm was used throughout.

1.2 Characterisation

NMR spectra were recorded by dispersing the sample (ca. 5-10 mg) in 0.7 mL of the desired deuterated solvent and acquired on a 2-channel Bruker AVIII 400 nanobay instrument running TOPSPIN 3 equipped with a 5 mm z-gradient broadband multinuclear probe. Attenuated Total Reflectance infrared (ATR-IR) spectra were recorded on an IRTracer-100 (Shimadzu) spectrometer. Ultraviolet-visible (UV-Vis) spectra were obtained using a UV-2401PC (Shimadzu) spectrometer. ICP-MS measurements were performed using a PerkinElmer NexION 2000B ICP-MS spectrometer. Samples were prepared for ICP-MS by digestion in 3.00 mL of concentrated (70%) HNO_3 at room temperature for > 24 h. Then, 0.2847 mL of the digested sample was added to deionised water (DI) to achieve a total volume of 15 mL. The [Gd^{3+}] calibration curve was prepared using SPS-SW2 standard (Spectrapure Standards, Oslo). A Malvern Zetasizer Nano with a 532 nm laser as the light source was employed for Dynamic Light Scattering (DLS) analyses. The samples for DLS were prepared by dispersing the nanoparticles in DI water, DI Water/EtOH, or 10 mM K_2SO_4. Barrett–Joyner–Halenda (BJH) nitrogen adsorption-desorption analyses were obtained using a Micromeritics TriStar II PLUS surface characterisation analyser. Thermogravimetric analysis (TGA) was performed using a METTLER TOLEDO micro and ultra-micro balances. MRI measurements were performed using an inversion recovery pulse sequence on a Spinsolve 60 T1 Magritek 1.4 T NMR machine (2 scans, acquisition time 1.6 s, repetition time of 10 s, max inversion time of 2 s and 11 steps). Samples were dispersed in the respective mediums 30 minutes prior to T_1 measurement. The measurements were recorded for 1.0x,

0.5x, and 0.25x dilutions of each sample, with the r_1 values calculated from the gradient of the resulting linear fit of a plot of $1/T_1$ versus the concentration of Gd (III) (as determined by ICP-MS).

1.3 Synthesis

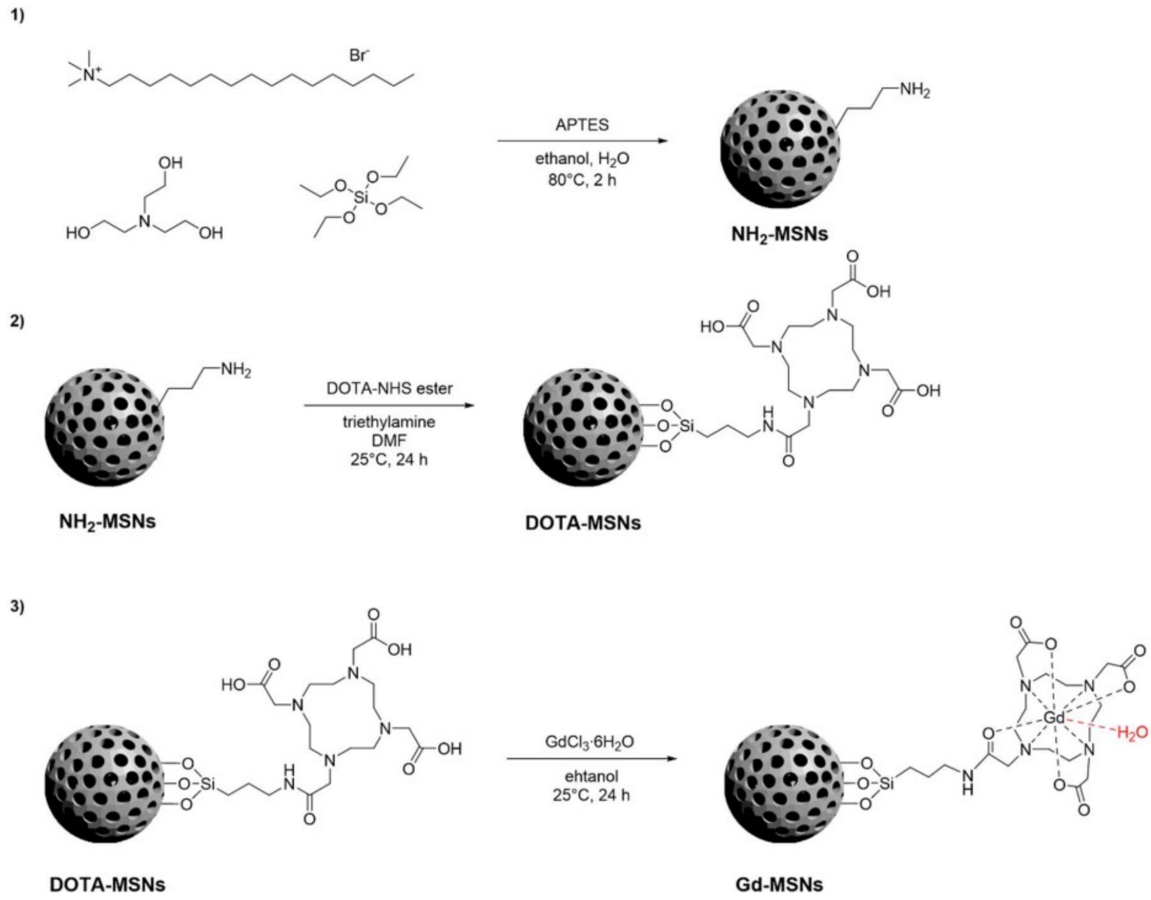

1.3 Schematised depiction of the synthetic steps required to produce Gd-MSNs.

1.3.1. Mesoporous Silica Nanoparticles Covalently Modified with Gd-DOTA (Gd-MSNs)[1]

0.3% NH_2-MSNs: The synthesis of **double-delay NH_2-MSNs** was performed as a combination of the syntheses for **short-delay** and **long-delay NH_2-MSNs** previously reported by our group.[1,2] Cetyl trimethylammonium bromide (CTAB, 0.645g, 1.77 mmol) and triethanolamine (TEOA, 1.029g, 6.9 mmol) were dissolved in 1.88 mL water and 16.20 mL ethanol. The mixture was stirred at 80 °C for 20 minutes to allow for micelle formation. Tetraethyl orthosilicate (TEOS, 1.156 mL, 5.18 mmol) was then added dropwise at 1 mL/min into the reaction mixture under stirring. After 10 minutes, 3-aminopropyltriethoxysilane (APTES, 2.6 µL) was added to the mixture. After 50 additional minutes of stirring, another 2.6 µL of APTES was added alongside 2.23 µL of TEOS. The reaction was then stirred for a total reaction time of 2h. The mixture was then cooled down to room temperature, whereupon the particles were collected via centrifugation (13500 rpm, 20 min). The particles were then purified

using centrifugation in ethanol (2x). 30 minutes of sonication in acidified EtOH (10 vol%) was then used to remove the CTAB template. The particles were washed two additional times with EtOH. The particles were then collected via centrifugation and dried under vacuum for 24h to yield the desired double delay aminated MSNs.

Gd-MSNs: 200 mg of dried **NH_2-MSNs** were dispersed in 15 mL DMF via sonication. Then, 5.25 μmol of DOTA-NHS ester and 150 μL of triethylamine were added to the reaction flask. The resultant mixture was stirred at r.t for 24 h. The nanoparticles were then collected by centrifugation at 13,500 rpm for 20 min and washed three times with EtOH to produce the desired DOTA modified MSNs (DOTA-MSNs). The obtained DOTA-MSNs were dispersed in 10 mL of EtOH to which 10 μmol of $GdCl_3.6H_2O$ was added. Then, vigorous stirring was carried out for 24 h at r.t. The Gd-doped nanoparticles (Gd-MSNs) were washed three times with EtOH and dried under vacuum.

Surface Aminated GdMSNs: 50 mg of **GdMSNs** were dispersed in a mixture of water and ethanol (5/2 mL) with 400 μL of ammonium hydroxide added. 40 μL of APTES was then added dropwise and the mixture stirred at RT for 24 hours. The particles were then collected by centrifugation and washed with EtOH three times. The particles were dried under vacuum at 50°C overnight.

Surface Carboxylated GdMSNs (COOH-GdMSNs): 40 mg of **Surface Aminated GdMSNs** were dispersed in 5 mL of anhydrous DMF to which 30 mg of succinic anhydride was added. The mixture was stirred at RT for 24 hours. The particles were collected by centrifugation and washed with EtOH, DMF, EtOH. The particles were dried under vacuum at 50 °C overnight.

AZO-GdMSNs: 30 mg of **COOH-GdMSNs** were dispersed in 5 mL anhydrous DMF and cooled to 0 °C. 10.88 mg of NHS and 18.4 mg of EDC*HCl were then added. The mixture was stirred for 30 mins at 0 °C, before being left to rise to RT and stirred for an additional 1.5 hours. 30.355 mg of AZO was then dissolved in 4 mL anhydrous DMF, and 40.4 μL of TEA added. The AZO solution was then added to the activated MSN dispersion and the mixture stirred at RT for 24 hours. DLS confirmed the lack of particle cross-coupling (through the absence of larger aggregates). The particles were collected by centrifugation and purified with EtOH, DMF, EtOH. The particles were dried under vacuum at 50°C overnight.

CPADB-AZO-GdMSNs: 37.24 mg of CPADB was dissolved in 5 mL anhydrous DMF at 0 °C. 18.4 mg of NHS and 33.0 mg of DCC were then added. The mixture was stirred for 30 mins at 0°C, and then left to rise to RT before being stirred for an additional 30 minutes. The precipitated dicyclohexylurea (DCU) was filtered out of solution. 30 mg of **AZO-GdMSNs** were then dispersed in 4 mL of anhydrous DMF, and 20.2 μL of TEA was added. The CPADB-NHS was added to the MSN dispersion and the mixture was

stirred at RT for 24 hours. The particles were collected by centrifugation and washed with DMF, EtOH, EtOH. The particles were left to dry under vacuum at 30 °C overnight.

CPADB-NHS: 139.69 mg of CPADB, 69.054 mg of NHS and catalytic DMAP were dissolved in 6 mL anhydrous DCM and cooled to 0 °C. 113.483 mg of DCC was then added to the mixture and stirred for 2h before being left to return to RT. The mixture was then cooled to -20 °C to encourage precipitation of DCU, which was then filtered out of the mixture. The solvent was removed under reduced pressure and the solid dried under vacuum at RT to yield CPADB-NHS. Calc. *m/z* 377.4, found *m/z* 377.32 [M+H+].

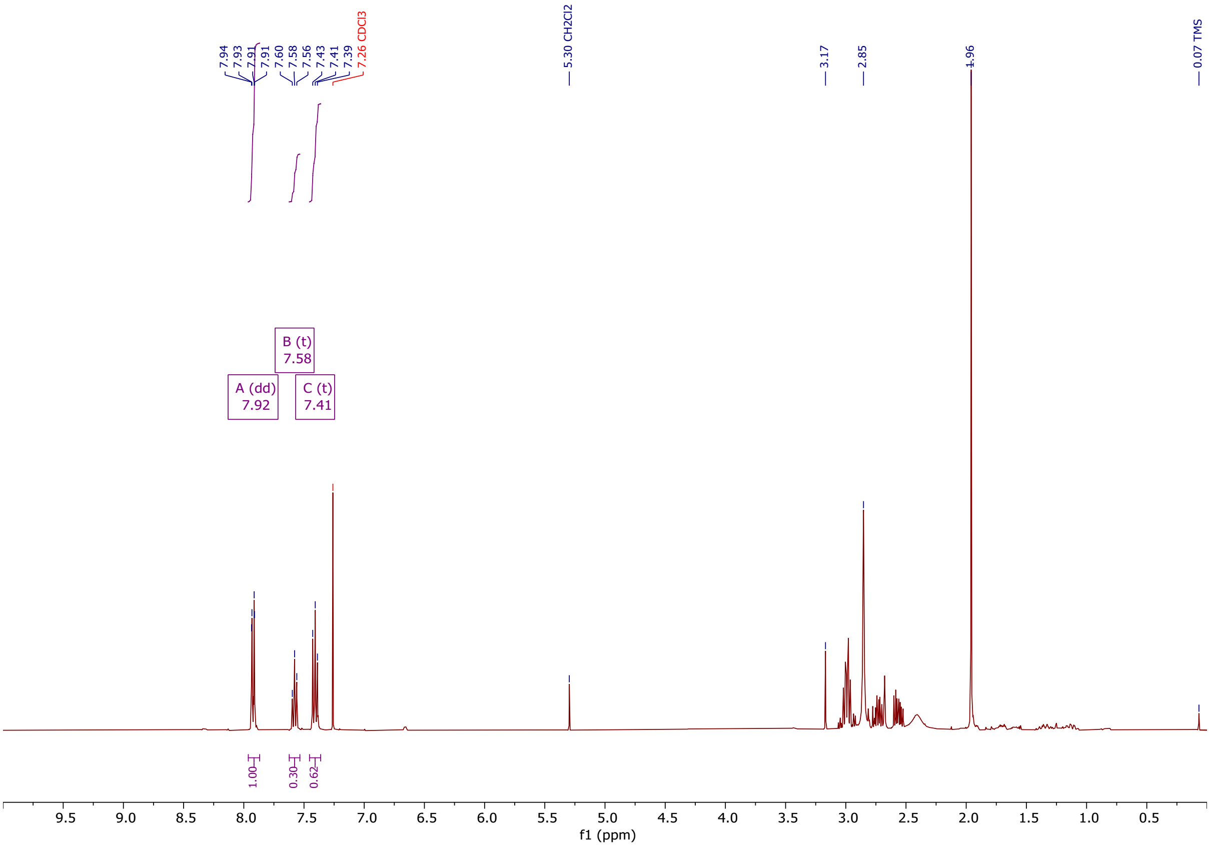

NMR Spectrum 1: CPADB-NHS

CPADB-AZO: 207.04 mg of CPADB-NHS was added to a mixture of 120.625 mg of 4-((4-Aminophenyl)diazenyl)benzoic acid and 104.5 µL of TEA in 15 mL of anhydrous DCM. The mixture was stirred for 24 hours at RT in the dark. The solvent was then removed under reduced pressure with the residue dissolved in 15 mL of ethyl acetate before being washed twice with 10 mL 0.1N HCl, and once with 15 mL brine. The organic layer was collected and dried over $MgSO_4$. The solvent was reduced by rotary evaporation. The concentrated residue was purified by silica gel column chromatography (95/5 DCM/EtOAc, 0.1% formic acid). Found *m/z* 531.25 [M+HCOO-]-.

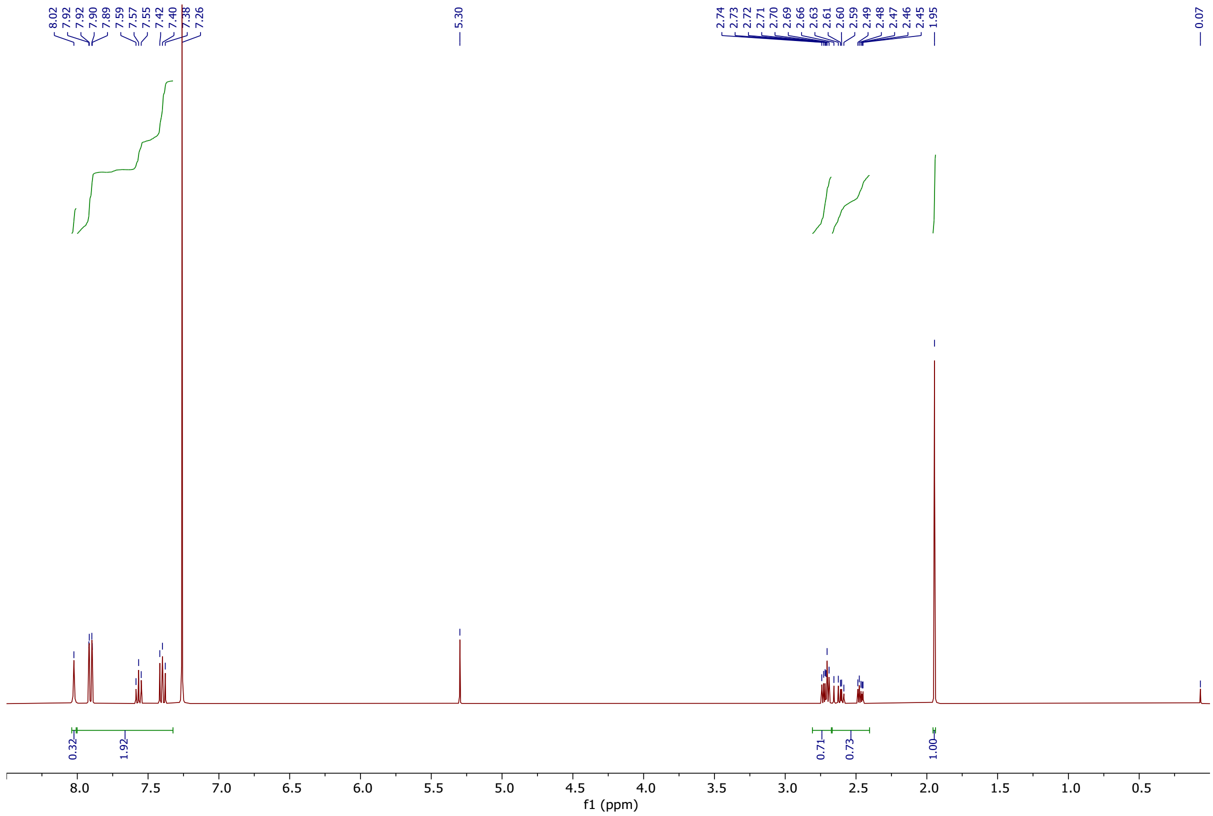

NMR Spectrum 2: CPADB-AZO

CPADB-AZO-Silane: 167.53 mg of CPADB-AZO was dissolved in 15 mL of anhydrous DCM with 46.035 mg of NHS and cooled to 0 °C. 75.655 mg of DCC was then added and the mixture stirred for 30 minutes, allowed to warm to RT, and stirred for an additional hour. A solution of 78 µL of APTES in 2 mL of anhydrous DCM was added to the mixture dropwise. The mixture was stirred for 24 hours in the dark. The solvent was then removed under a stream of nitrogen with the crude product triturated with 30 mL of cold anhydrous toluene. The impurities were filtered out and the solvent was removed by rotary evaporation. The crude residue was then purified by silica gel column chromatography with 1:2 dried EtOAc/Hexane to yield a viscous red oil. All reactions were carried out under an inert argon atmosphere.

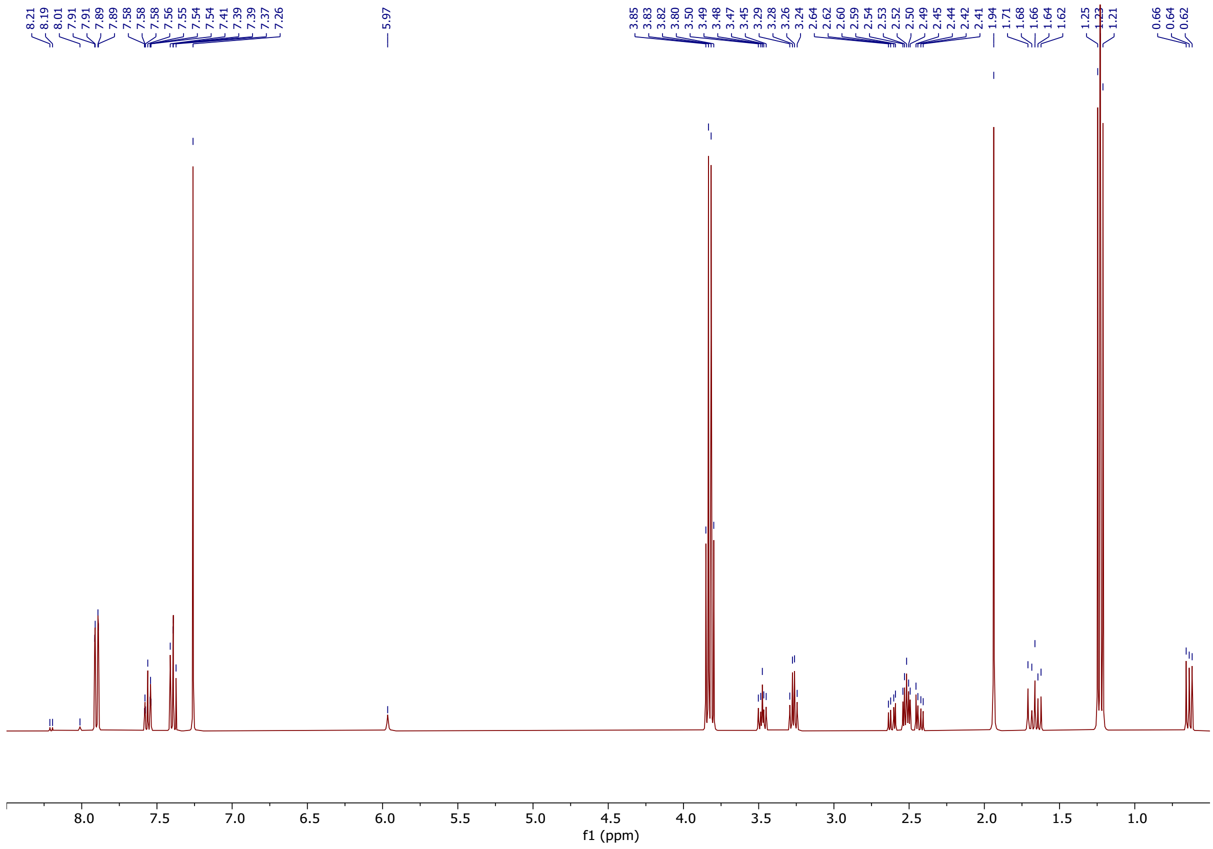

NMR Spectrum 3: CPADB-AZO-Silane

CPADB-AZO-GdMSNs (via silane): 50 mg of **GdMSNs** were dispersed in a mixture of EtOH and water (9:1). 100 µL of ammonium hydroxide were added with 35 µL of the **CPADB-AZO-Silane** subsequently added to the mixture, before being left to stir at RT in the dark for 24 hours. The particles were collected by centrifugation and washed with DMF, EtOH, EtOH. They were then dried under vacuum.

pDMAEMA-AZO-GdMSNs: 15 mg of **CPADB-AZO-GdMSNs** were suspended in 5 mL DMF. 0.594 mg of AIBN and 151.5 microliters of DMAEMA were added. The mixture was sealed and degassed with dry argon for 20 minutes. The mixture was stirred at 70 °C for 48 hours. The particles were collected by centrifugation and washed with DMF, EtOH, EtOH before being left to dry under vacuum.

pMAA-AZO-GdMSNs: 15 mg of **CPADB-AZO-GdMSNs** were suspended in 5 mL DMF. 0.594 mg of AIBN and 152.4 microliters of MAA were added. The mixture was sealed and degassed with dry argon for 20 minutes. The mixture was stirred at 70 °C for 48 hours. The particles were collected by centrifugation and washed with EtOH, EtOH, EtOH before being left to dry under vacuum.

HMSNs: A mixture of 25 mL EtOH, 0.7 mL water, and 0.9 mL ammonium hydroxide was prepared and mixed at RT for 20 minutes. 0.875 mL TEOS was then added dropwise and stirred for 24h. The particles were collected and washed with water, EtOH, EtOH before being suspended in 12.5 mL water.

A mixture of 3.41 mL EtOH, 25 mL water, 33.7 µL TEA, and 87.5 mg CTAB was prepared and mixed at 80 °C for 30 minutes. The particle suspension was then added and stirred for 30 minutes. 159.63 µL of TEOS was then added dropwise and the mixture was stirred for 3 hours. The particles were collected and washed with EtOH and then suspended in 12.5 mL water.

1925 mg of sodium carbonate was dissolved in 12.5 mL water and stirred at 50 °C for 15 minutes. The particle suspension was then added and the mixture stirred at 50 °C for 10 hours. The particles were collected and washed with water, EtOH, EtOH. The particles were dispersed in 20 mL acidified EtOH (3% HCl by volume) and refluxed at 80 °C for 6 hours, three times. The resulting HMSNs were washed with water, EtOH, EtOH, and dried under vacuum.

NH_2-HMSNs: 30 mg of **HMSNs** were dispersed in a mixture of EtOH and water (6/3 mL) and 480 µL of ammonium hydroxide was added. 49.14 µL of APTES was added dropwise and the mixture was stirred for 24 hours. The particles were collected and washed with EtOH x3. The particles were dried under vacuum at 50 °C.

COOH-HMSNs: 30 mg of **NH_2-HMSNs** were dispersed in 5 mL anhydrous DMF. 50 mg of succinic anhydride was then added and the mixture stirred for 24 hours. The particles were collected and washed with EtOH x3. The particles were dried under vacuum at 50 °C.

AZO-HMSNs: 15 mg of **COOH-HMSNs** were dispersed in 7 mL anhydrous DMF and was cooled to 0 °C. 16.32 mg of NHS and 27.6 mg of EDC*HCl were then added. The mixture was stirred for 30 mins at 0 °C, and was then allowed to rise to RT and stirred for an additional 1.5 hours. 45.53 mg of AZO was then dissolved in 4 mL anhydrous DMF, and 60.6 µL of TEA then added. The AZO solution was added to the activated HMSN dispersion with the mixture stirred at RT for 24 hours. DLS confirmed the lack of particle cross-coupling. The particles were collected by centrifugation and washed with EtOH, DMF, EtOH. The particles were dried under vacuum at 50 °C.

CPADB-AZO-HMSNs: 55.86 mg of CPADB was dissolved in 7 mL anhydrous DMF at 0 °C. 27.6 mg of NHS and 49.5 mg of DCC were then added. The mixture was stirred for 30 mins at 0 °C, before being left to RT and then stirred for an additional 30 minutes. The precipitated DCU was subsequently filtered out of solution. 25 mg of **AZO-HMSNs** were dispersed in 5 mL of anhydrous DMF, and 30.3 µL of TEA was added. The activated CPADB was then added to the HMSN dispersion and the mixture stirred for 24 hours. The particles were collected by centrifugation and washed with DMF, EtOH, EtOH. The particles were left to dry under vacuum.

pDMAEMA-AZO-HMSNs: 20 mg of **CPADB-AZO-HMSNs** were dispersed in 5 mL of 1:1 DMF/water with 30 mM Gd-DOTA. The dispersion was mixed at RT for 2 hours and then collected by centrifugation. 10

mg of these particles were dispersed in 5 mL DMF with 30 mM Gd-DOTA, to which 0.594 mg AIBN and 151.5 µL DMAEMA were added. The mixture was sealed and degassed with dry argon for 20 minutes, before stirring at 70 °C for 48 hours. The particles were collected by centrifugation and washed with DMF, EtOH, EtOH. The particles were then left to dry under vacuum.

2.0 Characterisation

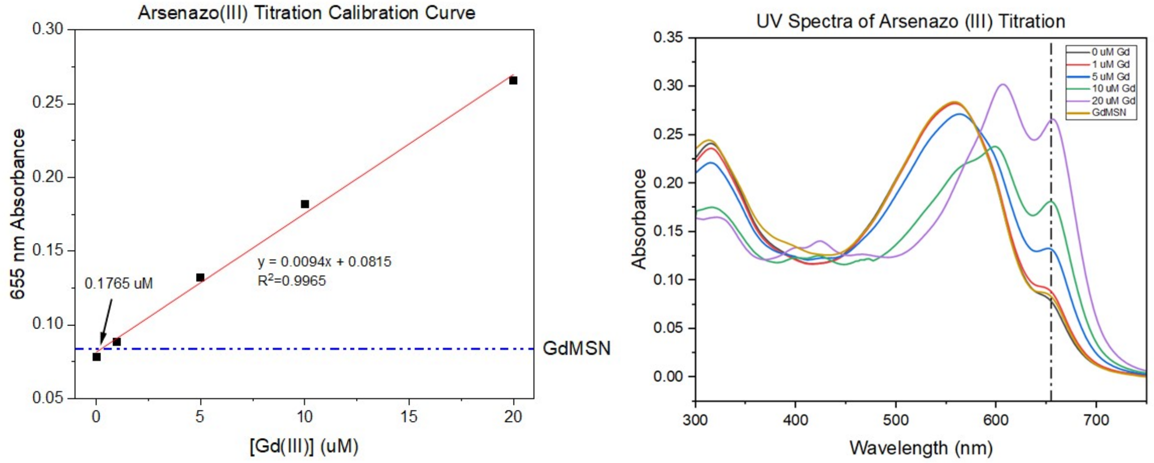

ESI 1A. Determination of the concentration of unchelated Gd(III) present within the native Gd-MSNs. A calibration curve of various Gd(III) concentrations using the UV-vis absorbance of the lanthanide sensitive dye Arsenazo(III) at 655 nm. Analysis of the titration revealed that there was 0.1765 µM of free Gd(III) present at a concentration of 1 mg/mL of particles (0.005% of total Gd(III)).

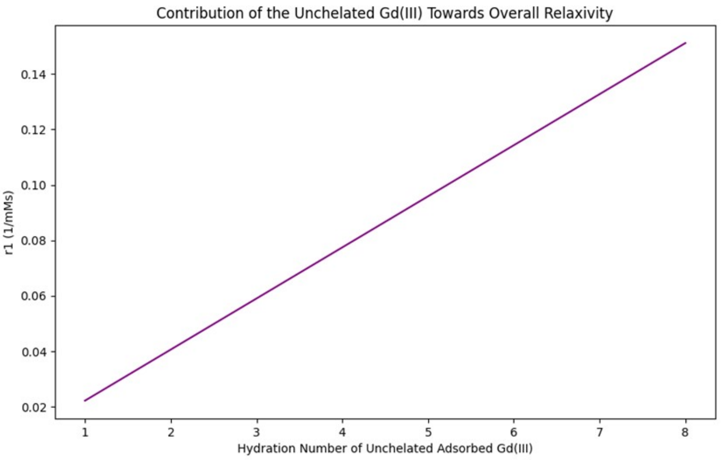

ESI 1B. Theoretical modelling of the relaxivity contributions of the unchelated Gd(III). Depending on the amount of vacant coordination sites on the ion (where 1 would be highly chelated by silica, and 9 would be complete solvation), the free ions may contribute between 0.02-0.14 mM^{-1} s^{-1} towards the overall relaxivity (27.6 mM^{-1} s^{-1}).

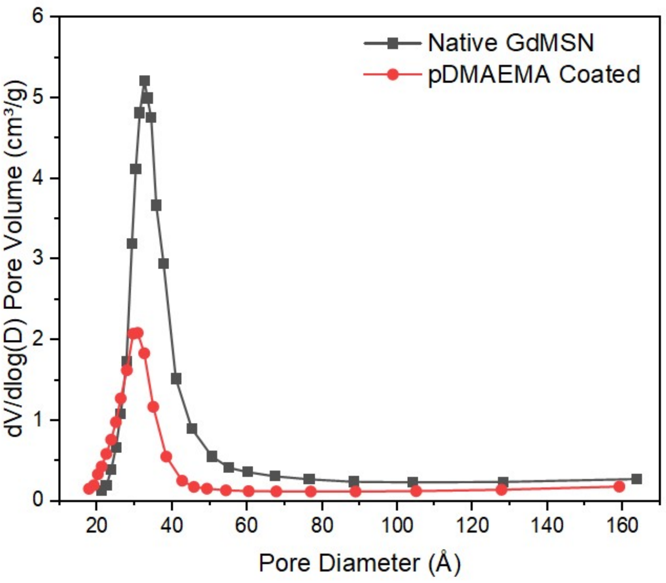

ESI 2. Barrett-Joyner-Halenda (BJH) pore size distributions of unmodified GdMSNs, and GdMSNs post-polymerisation with DMAEMA. Reduction in the total pore volume, and a 4-angstrom reduction in average pore size shows the effective blocking of pores by the grafted polymer.

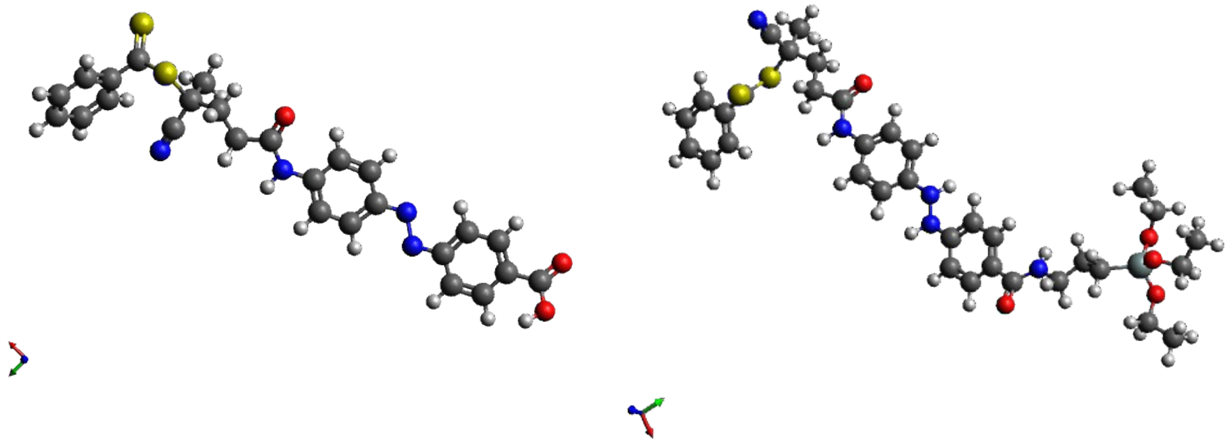

ESI 3. Optimised structures for CPADB-AZO (2.35 nm, left) and CPADB-AZO-Silane (3.12 nm, right) by DFT b3lyp method.

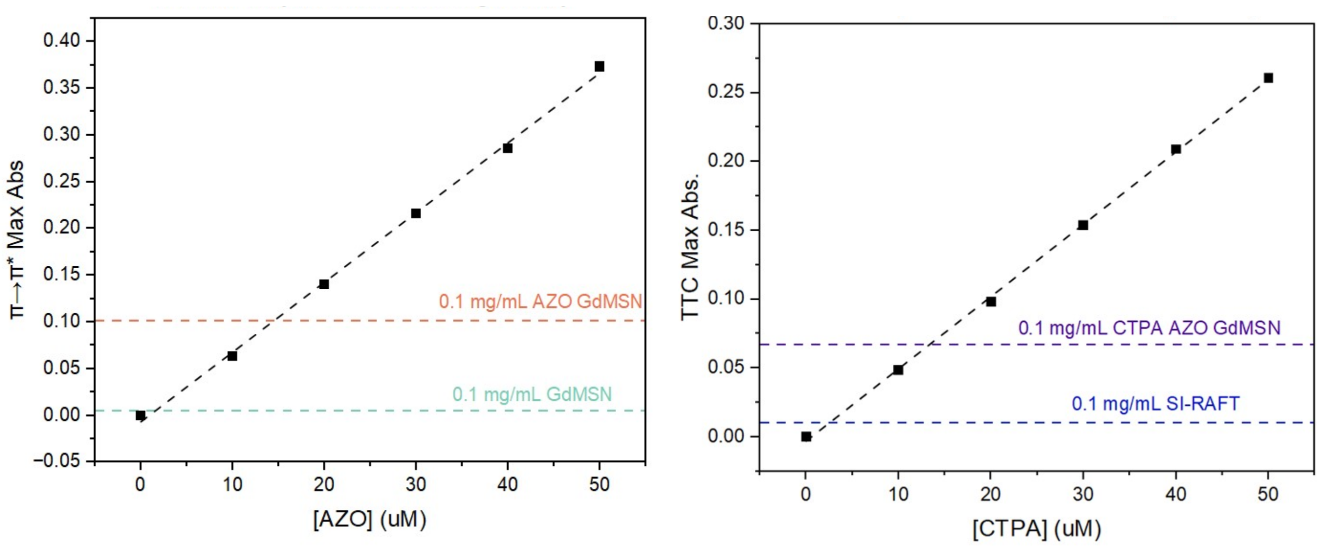

ESI 4. Calibration curves generated from solutions of the target molecule in DMF ranging from 0 - 50 μL, generated by UV-Vis. Max absorbance values of the target peaks obtained from spectra of nanoparticle dispersions concentrated at 0.1 mg/mL are inset into the spectra. SI-RAFT refers to a pDMAEMA-AZO-GdMSN. The max π -> π* absorbance of AZO is found at 371 nm. The max trithiocarbonate (or dithiobenzoate) absorption is found at 310 nm.

Particle	Grafting Density
AZO-GdMSN	1.871 groups per nm^2
CPADB-AZO-GdMSN	1.865 groups per nm^2

ESI 5. The grafting density of the two AZO and CPADB, calculated by dividing the absolute number of AZO or CPADB in 1 mL of solution over the total silica surface area in 0.1 mg of particles.[1] The grafting densities demonstrate a 99% conversion of AZO to CPADB-AZO by the employed amide coupling.

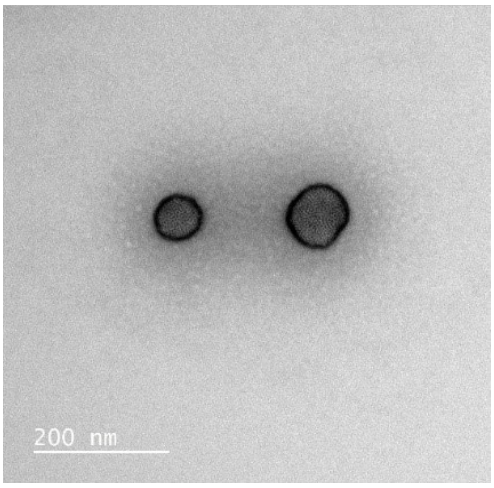

ESI 6. TEM imaging of pDMAEMA-AZO-GdMSNs stained with 2% uranyl acetate shows the characteristic dark outer shell of a polymer coated particle.

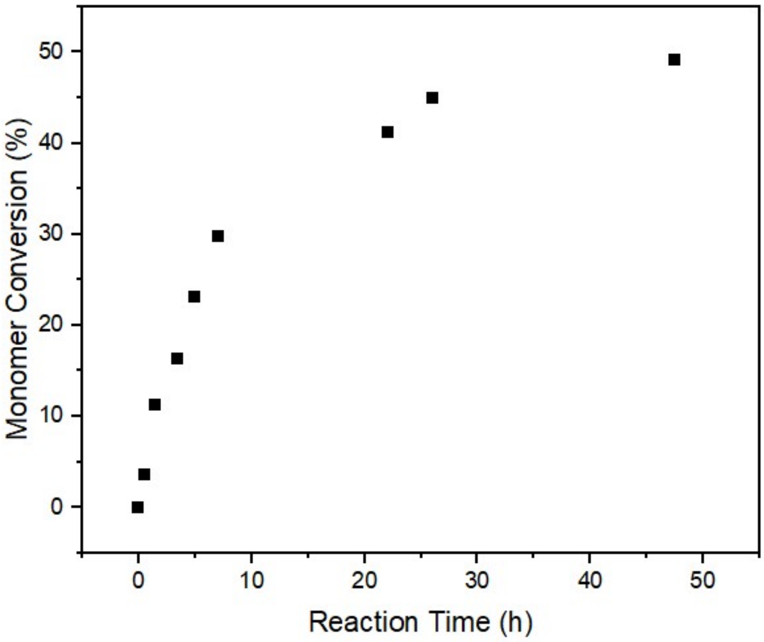

ESI 7. Monomer conversion plot for the SI-RAFT polymerisation of DMAEMA as monitored by ^{1}H NMR. The final conversion was 49.9% of the added monomer. The internal standard dimethyl sulfone was used to determine this value. The polymerisation demonstrates pseudo-first order kinetics until ~30% monomer conversion.

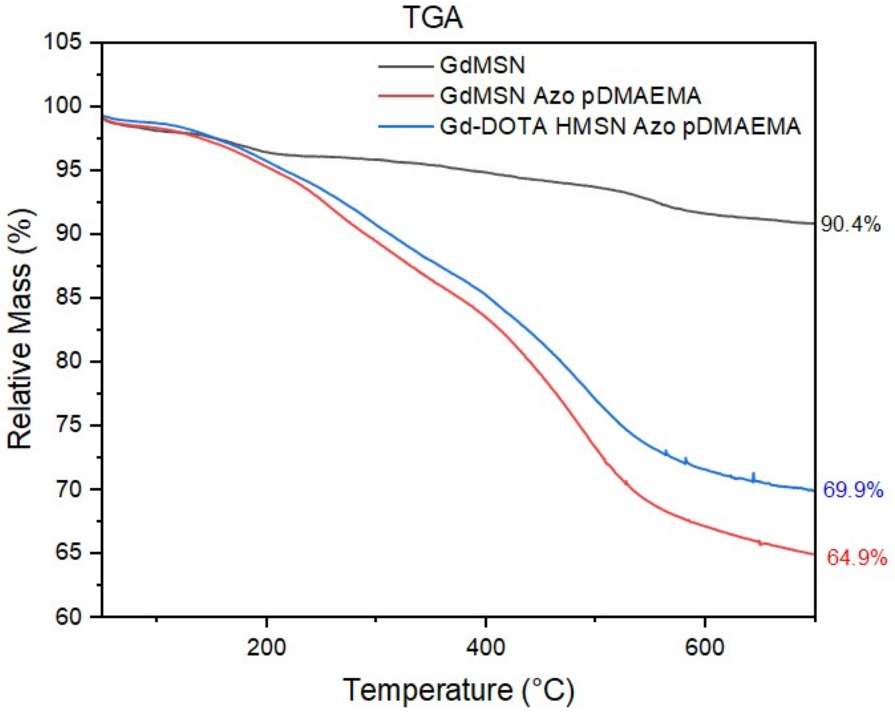

ESI 8. The thermogravimetric analysis (TGA) of GdMSNs, pDMAEMA-AZO-GdMSNs, and pDMAEMA-AZO-HMSNs. The weight losses at 700 °C for the particles are 9.6%, 35.1%, and 30.1%, respectively.

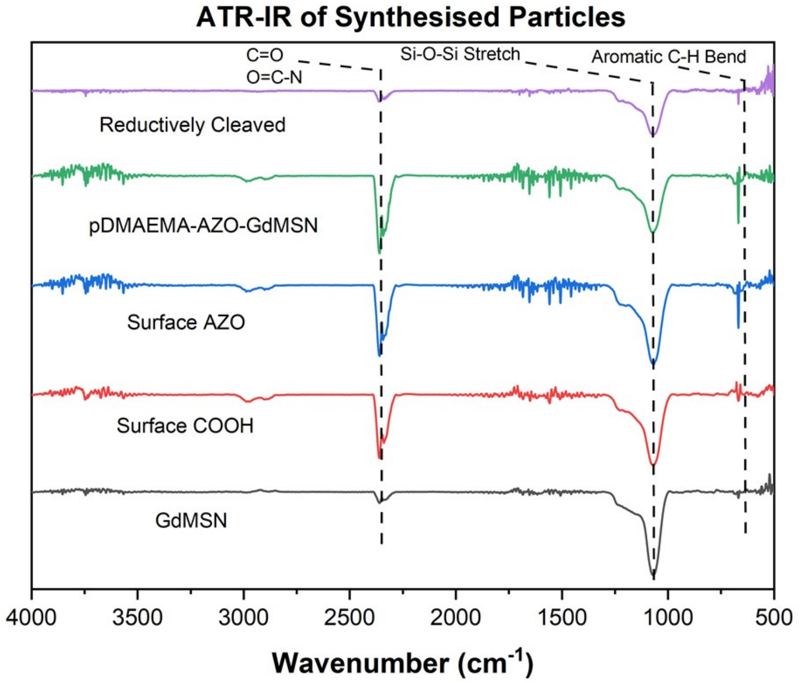

ESI 9. Attenuated total reflectance infrared spectroscopy (ATR-IR) spectra for the synthesised particles.

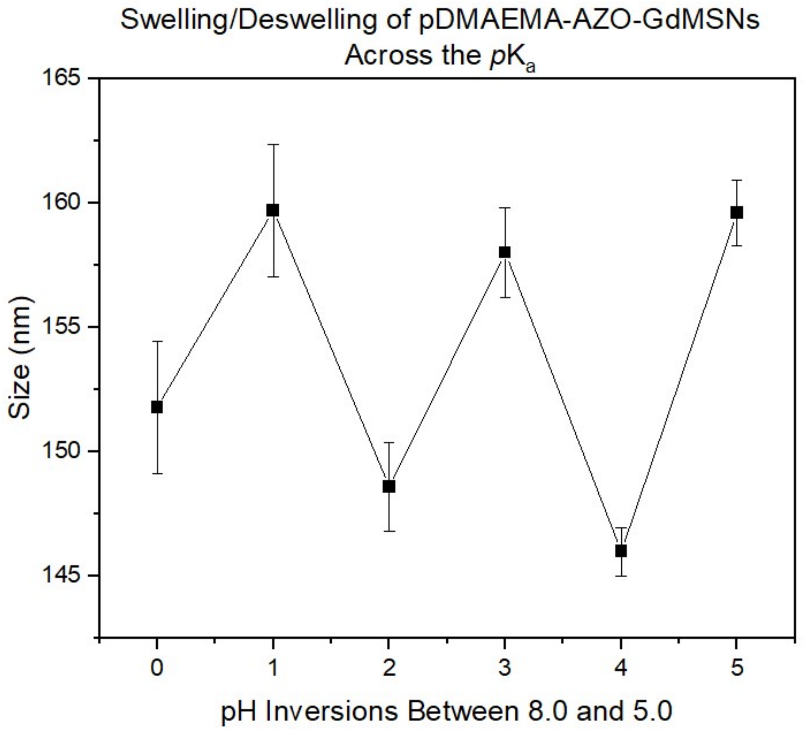

ESI 10. The reversible swelling of the pH-sensitive pDMAEMA coating of the particle, as the pH is inverted across the pK_a (~6.4).

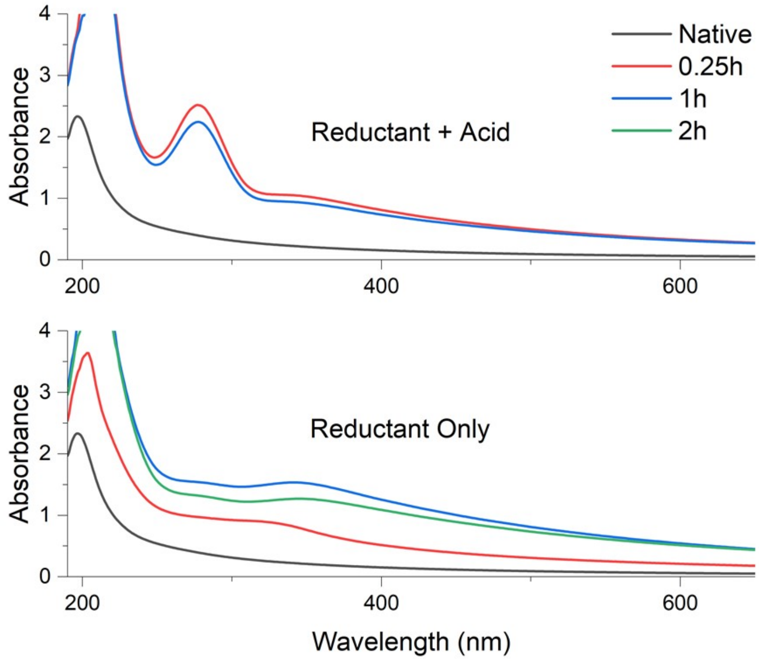

ESI 11. The UV-spectra of the particles exposed to reductant only or reductant and concurrent acid, demonstrating that both are required for the characteristic peak of the reduced cross-linker (aniline moiety) to appear at 290 nm, illustrating that reduction occurs rapidly under these conditions.

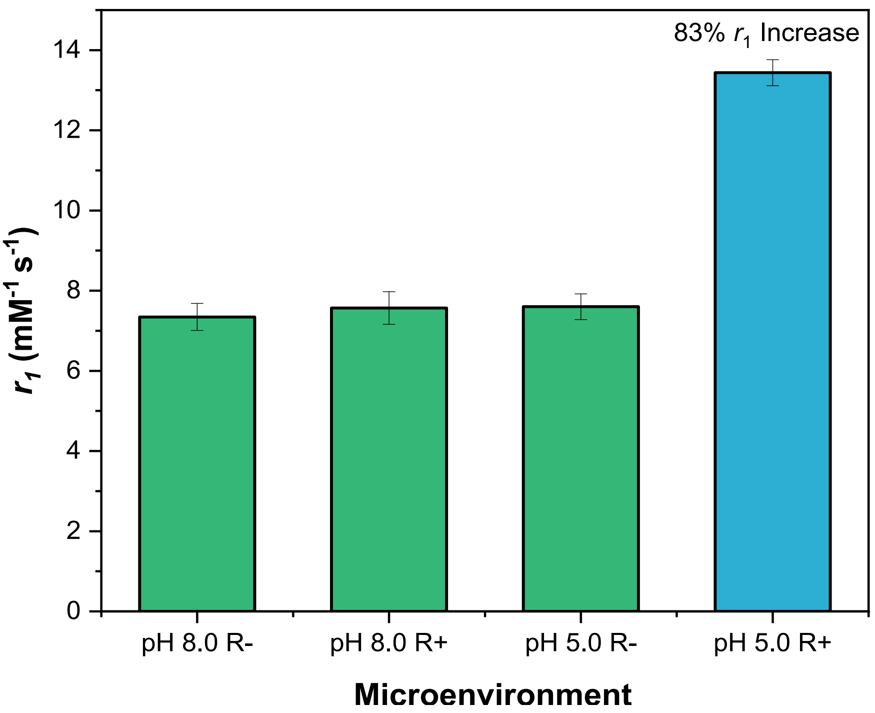

ESI 12. The impact of grafting density on the conformation of the bound polymer when exposed to a good solvent (i.e., water with pH < pK_a or THF). Low grafting density polymers (associated with the graft-to approach) adopt a globular conformation determined by their radius of gyration. This allows for steric gating between the reductant and cross-linker even when exposed to an acidic solution. Higher grafting polymers (associated with SI-RAFT) adopt the brush conformation, with significantly less polymer localised to the particle surface.

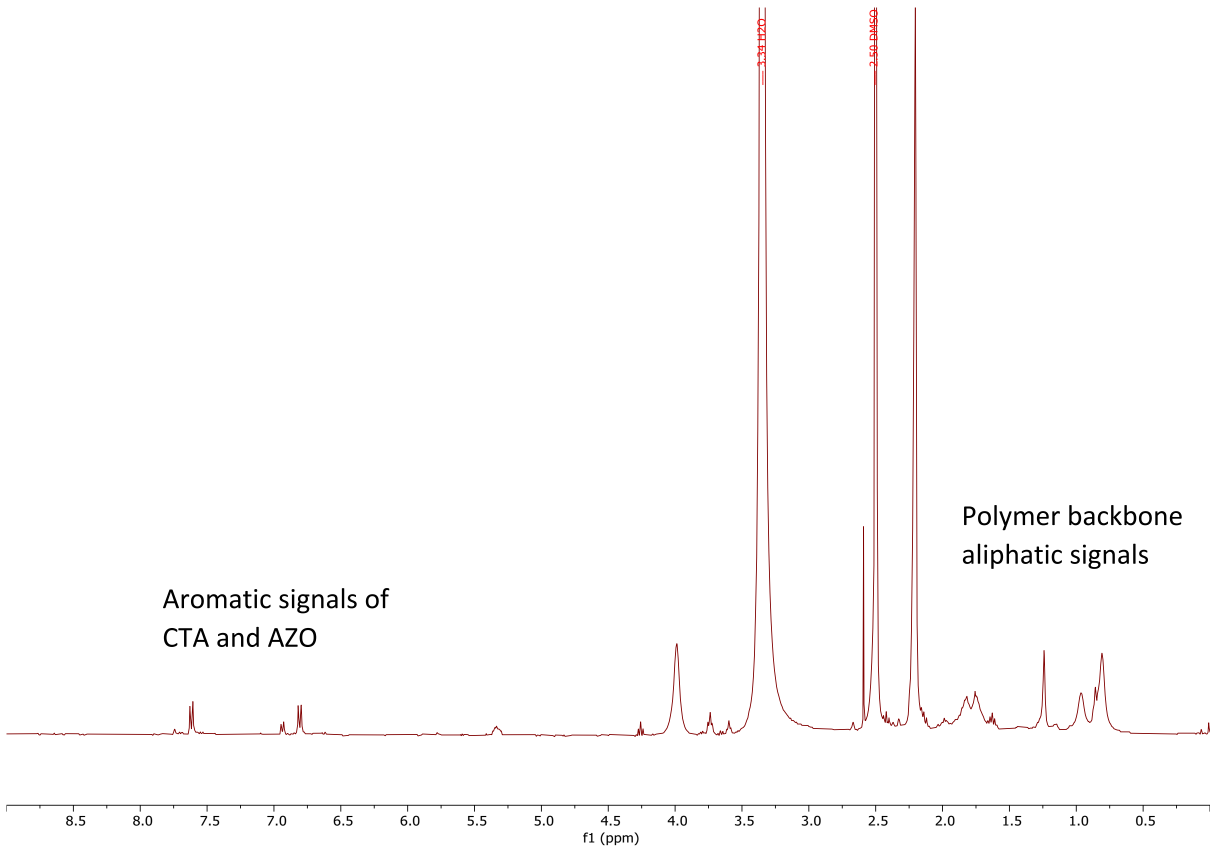

ESI 13. The ^1H NMR spectrum of the residue collected from a typical polymer cleavage reaction. The spectrum shows the characteristic pDMAEMA peaks, as well as aromatic signals resulting from the CTA and reduced aniline derivative terminating the polymer.

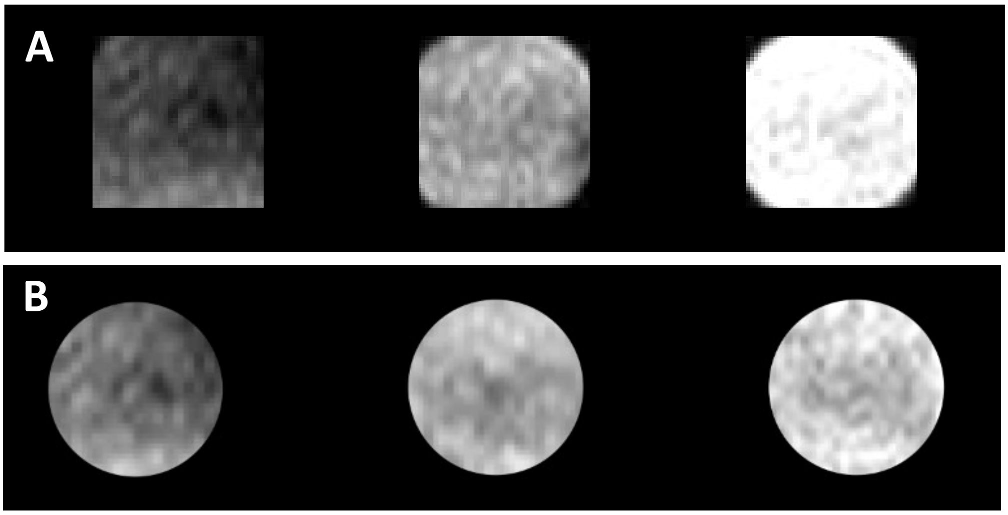

ESI 14. Raw phantom MRI images. A) pDMAEMA-AZO-GdMSNs with pure water on the left, pH 8.0 R- in the middle, and pH 5.0 R+ to the right. B) pDMAEMA-AZO-HMSNs with pure water on the left, pH 8.0 R- in the middle, and pH 5.0 R+ to the right.

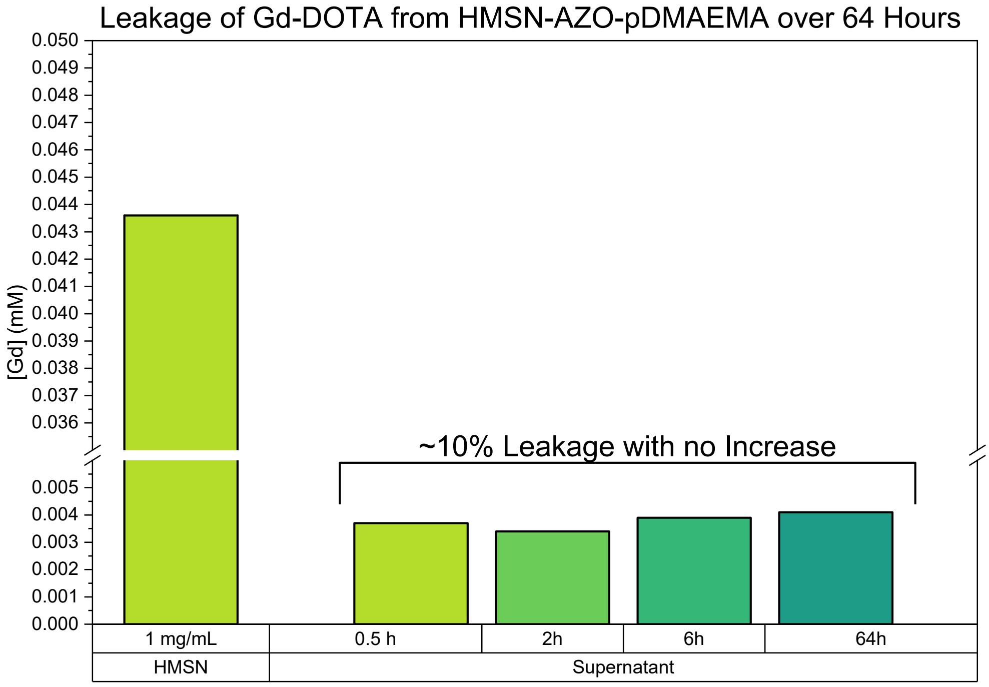

ESI 15. Leakage of Gd-DOTA from the pDMAEMA-AZO-HMSNs in pH 8.0 R- over 64 hours. Aliquots were collected by centrifuging out the particles at 13500 RPM and collecting a 300 µL aliquot for ICP-MS. The particles were immediately redispersed by sonication. The initial 10% "leakage" can be attributed to Gd-DOTA weakly adsorbed to the polymer brush, with no additional cargo being released prior to dual-stimuli exposure.

ESI 16. Analysis of Solomon-Bloembergen-Morgan (SBM) Theory[3-5]:

The observed longitudinal relaxivity value can be separated into the sum of its inner sphere (IS), second sphere (SS) and OS contributions (OS) (Eq. 1):

$$r_i = r_{i,IS} + r_{i,SS} + r_{i,OS}; \qquad i = 1, 2 \qquad (1)$$

with $i = 1, 2$ representing the longitudinal and transversal contributions respectively. The IS contribution arises from the nuclear spin residing in water molecules occupying the first coordination sphere of the paramagnetic ion and is summarised by the theory of Solomon, Bloembergen, and Morgan (SBM theory), as shown in Equation 2:

$$r_{i,IS} = \frac{q/[H_2O]}{T_{im} + \tau_m}; \qquad i = 1, 2,$$

(2)

where:

q is the number of bound IS water molecules ($q = 1$);

$[H_2O]$ is the water concentration;

T_{im} is the relaxation time of the water bound to the metal ($i = 1, 2$);

τ_M is the residence lifetime of the IS water molecules ($\tau_M = 15\ ns$).

From modified SBM theory, the IS longitudinal relaxation times, T_{1m}, can be expressed as:

$$\frac{1}{T_{1,DD}} + \frac{1}{T_{1,SC}} = \frac{2}{15}\frac{C}{r_{GdH}^6}\left[\frac{7\tau_{c2}}{1+\omega_s^2\tau_{c2}^2} + \frac{3\tau_{c1}}{1+\omega_I^2\tau_{c1}^2}\right] + \frac{2}{3}S(S+1)\left(\frac{2\pi A}{h}\right)^2\left[\frac{\tau_e}{1+\omega_s^2\tau_{e2}^2}\right];$$

(3)

where:

the constant C is given by $C = \gamma_I^2 g^2 \mu_B^2 \left(\frac{\mu_0}{4\pi}\right)^2 S(S+1)$;

and:

γ_I is the proton gyromagnetic constant ($\gamma_I = 2.675 \times 10^8\, T^{-1}\, s^{-1}$);

g is the electronic g-factor ($g = 2$);

S is the total electron spin of the material ion ($S = 7/2$ for Gd^{3+});

μ_B is the Bohr magneton ($\mu_B = 9.274 \times 10^{-24}\, J\, T^{-1}$);

μ_0 is the vacuum permeability ($\mu_0 = 1.257 \times 10^{-6}\, N\, A^{-1}$);

r_{GdH} is the distance between the metal ion and proton ($r_{GdH} = 0.31\, nm$);

ω_I and ω_S are the angular proton and electronic Larmor frequencies (with $\omega_S \approx 658\, \omega_I$, $\omega_I = \gamma_I B$, and B the magnetic field strength);

A is the hyperfine coupling constant (in J);

$T_{1,DD}$ and $T_{1,SC}$ are the relaxation times for the dipole-dipole and scalar relaxation mechanism between the water protons and the paramagnetic centre. The scalar contribution is often neglected and deemed to be negligible.

The dipole-dipole, τ_{c1} and τ_{c2}, and scalar, τ_e, correlation times are defined as:

$$\tau_{ci} = \left(\tau_R^{-1} + \tau_m^{-1} + T_{ie}^{-1}\right)^{-1}; \qquad (4)$$

$$\tau_{ei} = \left(\tau_m^{-1} + T_{ie}^{-1}\right)^{-1}; \qquad (5)$$

where $i = 1, 2$ is associated with T_1 or T_2 relaxation respectively.

τ_R denotes the tumbling rate of the complex;

T_{ie} are given by:

$$\frac{1}{T_{1e}} = \frac{1}{25}\Delta^2\tau_v[4S(S+1)-3]\left[\frac{1}{1+\omega_s^2\tau_v^2} + \frac{4}{1+4\omega_s^2\tau_v^2}\right]; \quad (6)$$

$$\frac{1}{T_{2e}} = \frac{1}{25}\Delta^2\tau_v[4S(S+1)-3]\left[\frac{5}{1+\omega_s^2\tau_v^2} + \frac{2}{1+4\omega_s^2\tau_v^2} + 3\right]; \quad (7)$$

where:

Δ^2 is the mean square zero field splitting (ZFS) energy ($\Delta^2 = 4.6 \times 10^{19}\ s^{-2}$);

τ_v is the correlation time for splitting ($\tau_v = 14$ ps);

Internal motion, where there are differences between the slow global motion of the supporting scaffold (*e.g.*, inorganic nanoparticle or polymer micelle) and faster local motion due to internal flexibility, can also be modelled. This was originally reported by Lipari and Szabo and involves approximating the spectral density function as:

$$j(\omega) = \frac{F^2 3\tau_{cG1}}{(1+\omega_I^2\tau_{cG1}^2)} + \frac{(1-F^2)3\tau_{cL1}}{(1+\omega_I^2\tau_{cL1}^2)}; \quad (8)$$

where:

$$\tau_{cG1} = \left(\tau_{RG}^{-1} + \tau_M^{-1} + T_{1e}^{-1}\right)^{-1}; \quad (9)$$

$$\tau_{cL1} = \left(\tau_{cG1}^{-1} + \tau_{RL}^{-1}\right)^{-1}; \quad (10)$$

and:

F denotes an order parameter that takes a value between 0 and 1;

τ_{RG} is the global correlation time;

τ_{RL} is the local correlation time;

τ_{cL1} the local correlation time which accounts for fast local motion ($1/\tau_{cL1} = 1/\tau_{cG1} + 1/\tau_{RL}$).

Depending on the value of F^2 the motion is either governed by the global relaxation time ($F^2 = 1$) or the fast local motion ($F^2 = 0$).

The OS contribution to the longitudinal relaxivity, arising from the translational diffusion of water molecules in the vicinity of the paramagnetic ion, can be summarised by the following equation as initially described by Freed:[6, 7]

$$r_{1,OS} = \frac{1}{c}\left(\frac{1}{T_1}\right)_{OS} = \left(\frac{32\pi}{405}\right) C_{DD} \frac{N_A}{aD} Re\left[3J(\omega_I) + 7J(\omega_S)\right]; \quad (11)$$

c is the concentration (in mM) of the paramagnetic ions in solution, a the distance between the metal ion and proton (estimated at 0.5 nm for OS), D the sum of the diffusion coefficients of bulk water and the complex, and N_A Avogadro's constant ($N_A = 6.023 \times 10^{23}$). ω_I and ω_S are the angular proton and electronic frequencies ($\omega_S = 658\omega_I$, $\omega_I = \gamma_I B$, with B the magnetic field strength), and A the hyperfine coupling constant (in J). Re is the real part of the spectral density function $J(\omega)$ which is given by:

$$J(\omega) = \left[4 + \left(i\omega\tau_D + \frac{\tau_D}{T_{1e}}\right)^{1/2}\right] \Big/ \left[4 + 4\left(i\omega\tau_D + \frac{\tau_D}{T_{1e}}\right)^{1/2} + \frac{16}{9}\left(i\omega\tau_D + \frac{\tau_D}{T_{1e}}\right) + \frac{4}{9}\left(i\omega\tau_D + \frac{\tau_D}{T_{1e}}\right)^{3/2}\right] \quad (12)$$

with τ_D ($\tau_D = a^2/D$) representing the diffusion correlation time. All other quantities have been prior defined (*vide supra*).

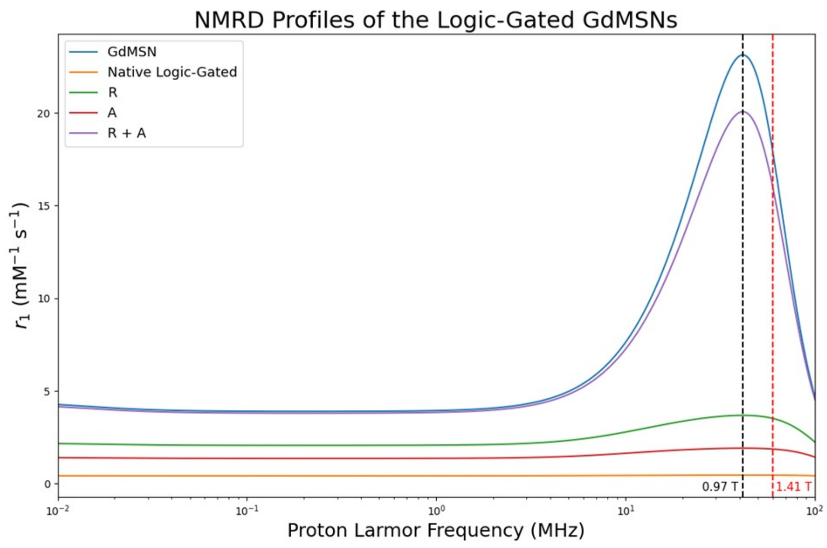

ESI 16.1. Inner sphere (IS) nuclear magnetic resonance dispersion (NMRD) profiles of the pDMAEMA-AZO-GdMSNs under each condition, as well as the native GdMSN. At the measured 1.41T, there is a 38.73x enhancement of the inner sphere relaxivity when comparing the native particle to the particle under acidic and reducing conditions. The optimal IS enhancement would be 49.88x at 0.97T. Native conditions are at pH > pK_a of pDMAEMA, A is exposure to solely acidic conditions (pH < pK_a), R is exposure to solely reductant, and R + A is concurrent stimuli exposure.

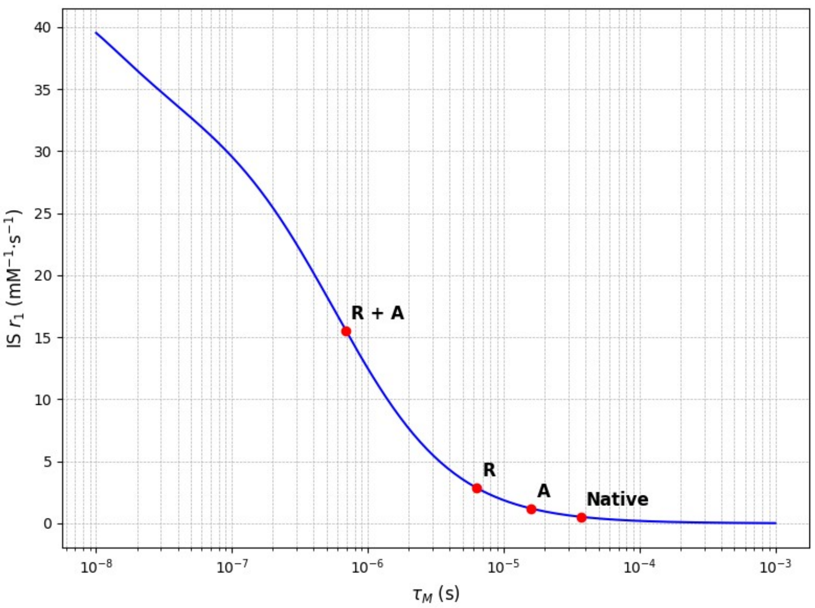

ESI 16.2. The dependence of inner sphere relaxivity on the residence time of water, with the four measurement conditions marked on the curve. Native conditions are at pH > pK_a of pDMAEMA, A is

exposure to solely acidic conditions (pH < pK_a), R is exposure to solely reductant, and R + A is concurrent stimuli exposure.

Mass of one MSN:

$$\frac{4}{3}\pi * r^3 * \rho = 0.302 \text{ fg}$$

Mass of polymer brush (1.86 CTA per nm^2) :

$$1.86 * 4\pi * r^2 * D_P * 157.21 = 0.122 \text{ fg}$$

%Weight of polymer brush:

$$\frac{0.122}{0.302 + 0.122} = 28.77\%$$

ESI 17. Calculations for the weight percent of the polymer brush.

References:

1. D. Yuan, C. M. Ellis, F. E. Mózes and J. J. Davis, Chemical Communications, 2023, 59, 6008–6011.
2. J. J. Davis, W. Y. Huang and G. L. Davies, J Mater Chem, 2012, 22, 22848–22850.
3. P. Caravan, C. T. Farrar, L. Frullano and R. Uppal, Contrast Media Mol Imaging, 2009, 4, 89–100.
4. J. A. Peters and K. Djanashvili, European Journal of Inorganic Chemistry, 2012, 2012, 1961–1974.
5. G. Lipari and A. Szabo, Journal of the American Chemical Society, 1982, 104, 4546–4559.
6. J. H. Freed, The Journal of Chemical Physics, 1978, 68, 4034–4037.
7. L. P. Hwang and J. H. Freed, The Journal of Chemical Physics, 1975, 63, 4017–4025.
